# A High-Performance Thermal Charging Cell with High Power Density and Long Runtime Enabled by Zn^2+^ and NH_4_^+^ Co-insertion

**DOI:** 10.1007/s40820-025-02011-9

**Published:** 2026-01-05

**Authors:** Zhiwei Han, Shengliang Zhang, Helang Huang, Jing Wang, Hui Dou, Tianran Zhang, Xiaogang Zhang

**Affiliations:** 1 Jiangsu Key Laboratory of Electrochemical Energy Storage Technologies, College of Material Science and Technology, Nanjing University of Aeronautics and Astronautics, Nanjing, 211106 People’s Republic of China; 2 College of Material Science and Opto-Electronic Technology, University of Chinese Academy of Sciences, Beijing, 100049 People’s Republic of China

**Keywords:** Thermal charging cells, Zn^2+^/NH_4_^+^ hybrid ions, Low-grade heat conversion and storage, High power density, Hydrated V_2_O_5_

## Abstract

Zn-based thermal charging devices, utilizing the synergistic effect of ion thermoextraction and thermodiffusion, are able to efficiently convert thermal energy into electrical energy and storage in the devices, making them a highly promising technology for low-grade heat recovery and utilization. However, the low output power density and energy conversion efficiency resulted by the slow diffusion kinetics of Zn^2+^ hinder their development. Herein, we present a high-performance thermal charging cell design using Zn^2+^/NH_4_^+^ hybrid ion electrolyte, which not only maintains the high output voltage of the Zn-based thermoelectric system, but also significantly enhances the output power density due to the fast diffusion kinetics of NH_4_^+^. Based on this strategy, the thermal charging cell displays a high thermopower of 12.5 mV K^−1^ and an excellent normalized power density of 19.6 mW m^−2^ K^−2^ at a temperature difference of 35 K. The Carnot-relative efficiency is as high as 12.74%. Moreover, it can operate continuously for over 72 h when the temperature difference persists, achieving a balance between thermoelectric conversion and output. This work provides a simple and effective strategy for the design of high-performance thermal charging cells for low-grade heat conversion and utilization.

**Supplementary Information:**

The online version contains supplementary material available at 10.1007/s40820-025-02011-9.

## Highlights

- The hybrid ion system strategically combines the high-voltage characteristics of Zn^2+^ redox with the exceptionally fast kinetics of NH_4_^+^, significantly boosting thermoelectric performance for low-grade heat harvesting.
- The Zn^2+^/NH_4_^+^ co-insertion/thermoextraction mechanism is elucidated, where NH_4_^+^ exhibits exceptionally fast migration due to its unique hydrogen bonding diffusion behavior.
- The device achieves a record 19.6 mW m⁻^2^ K⁻^2^ normalized power density with 72 h continuous operation, demonstrating strong application potential

**Supplementary Information:**

The online version contains supplementary material available at 10.1007/s40820-025-02011-9.

## Graphical abstract

**Figure:**
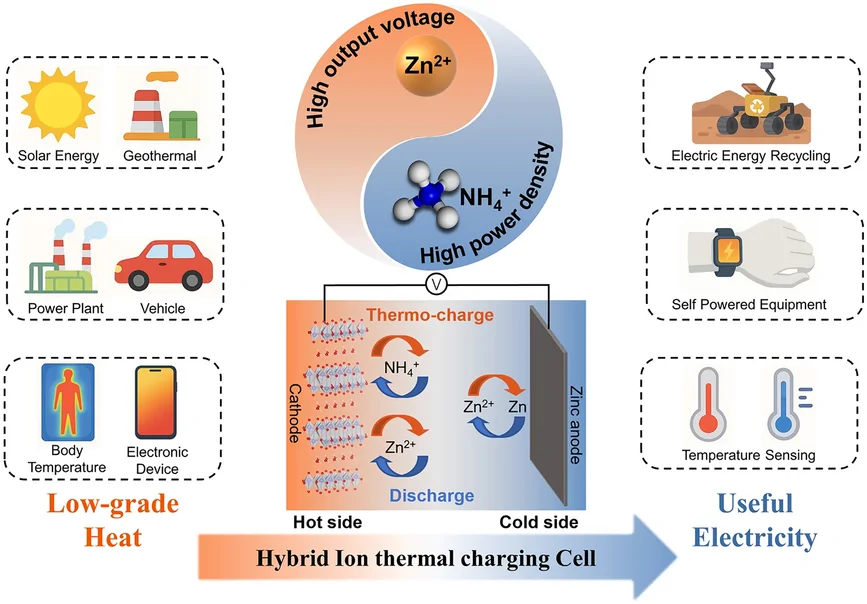

## Introduction

Low-grade heat (< 100 °C) is generated in daily life and industrial production of human society, which is often neglected and wasted because it is difficult to utilize efficiently [1–3]. Conventional solid-state thermoelectric devices (s-TEs) based on the Seebeck effect can realize the direct conversion of heat to electricity [4]. However, the relatively low Seebeck coefficient (~ µV K^–1^) still limits its practical application in areas such as Internet-of-Things (IoTs) sensors and wearable devices [5–7]. The emerging ionic thermoelectric devices (i-TEs), with a thermopower on the order of mV K^–1^, have overcome the critical limitation of insufficient energy conversion efficiency of s-TEs, offering new possibilities for the utilization of low-grade heat and source-free power supply for low-power devices [8–10].

During last decade, substantial progress has been made in the area of i-TEs. The thermopower of i-TEs has effectively improved by regulating the entropy difference of thermally driven redox reactions or designing nanostructure electrode materials [11–16]. However, the energy conversion efficiency remains relatively low. Moreover, additional energy storage units are often required for practical applications, thereby increasing the complexity of system integration [17–19]. To address these challenges, our group has developed novel zinc-ion thermal charging cells (ZTCCs) combining zinc-ion batteries with the ionic thermoelectric system, which not only improve the thermoelectric conversion efficiency but also integrate energy conversion with energy storage into a single system [20]. Through the thermoextraction of Zn^2^⁺ (its thermal-driven ejection from the cathode lattice) and its subsequent thermodiffusion (the concentration-gradient-driven diffusion in the electrolyte), coupled with the plating/stripping process on the Zn metal anode, the ZTCCs realized the high-efficiency conversion and storage of low-grade heat into electrical energy. By rationally designing the structure of the VO_2_ cathode material, a thermopower of 12.5 mV K^–1^ and an energy conversion efficiency of 0.95% (7.25% of the relative efficiency of Carnot) were obtained for the ZTCCs [21]. Furthermore, by employing a V_2_O_5_ cathode material with a higher ionic diffusion rate, the thermopower of the ZTCCs was further enhanced to 23.4 mV K^–1^ [22]. In addition, Cui et al. introduced the concept of zinc-ion hybrid supercapacitors into ionic thermoelectric systems, selecting a common adsorption-type cathode (commercial activated carbon YP-80F) to match with Zn metal anode, and assembled a new type of zinc-ion thermally charged supercapacitor (ZTCSCs) [23]. Specifically, by utilizing the reversible adsorption and desorption processes of electrolyte ions to achieve rapid energy conversion and storage, they obtained a thermopower of 15.1 mV K^−1^ and a normalized power density of 0.22 mW m^−2^ K^−2^ under a 30 K temperature difference. Further, the researchers also introduced redox species (I^–^/I_3_^–^) into the ZTCSCs. The thermopower and normalized power density were enhanced to 19.2 mV K^−1^ and 0.44 mW m^−2^ K^−2^ by the synergistic action of multiple mechanisms. Overall, Zn-based thermoelectric systems represent a sustainable and economically viable solution for efficient harvesting and storage of low-grade thermal energy.

Despite the fact that the addition of redox couples and the optimizations in electrode materials have effectively enhanced the thermopower of Zn-based thermal charging devices, the relatively large ionic radius of Zn^2+^ and its strong electrostatic interactions with the cathode material framework lead to sluggish ion diffusion kinetics [24–27]. This results in a relatively small enhancement in output power density and thermoelectric conversion efficiency of the i-TEs. Over the past few years, the nonmetallic carrier NH_4_^+^ has received increasing attention because of its lightweight and small radius of hydrated ion. Owing to its unique hydrogen bond diffusion mechanism, NH_4_^+^ exhibits faster diffusion kinetics than most metal ions [27–29]. In recent years, the concept of hybrid-ion batteries has gained increasing attention [30–34]. Typically, Li et al. developed a Zn^2^⁺/NH₄⁺ hybrid-ion battery, exploiting the "gap-filling" mechanism of NH₄⁺ to substantially improve the utilization efficiency of cathode materials [35]. This innovation effectively enhanced the overall ion migration rate, thereby achieving remarkable improvements in rate performance and cycling stability. Our previous work has verified that NH_4_^+^ has a good potential for application in ionic thermoelectric systems [36]. If NH_4_^+^ can be incorporated as a charge carrier into the Zn-based thermoelectric system, it is expected to address the issues of insufficient thermoelectrochemical reaction kinetics, while maintaining the high output voltage and thermopower performance [35].

Herein, we designed and assembled novel Zn^2+^/NH_4_^+^ hybrid ion thermal charging cells (HTCCs) with high power density and long runtime based on hydrated vanadium pentoxide (HVO) cathode, Zn metal anode, and Zn^2+^/NH_4_^+^ hybrid ion electrolyte. A variety of ex situ characterizations and electrochemical measurements have demonstrated the co-insertion/thermoextraction mechanism of NH_4_^+^ and Zn^2+^. In addition, first principles calculations also reveal that NH_4_^+^ can achieve rapid diffusion through hydrogen bonding interactions with the interlayer crystalline water of the cathode material. As designed, the HTCCs show a high output voltage of ~ 1.08 V and a high thermopower of 12.05 mV K^–1^. Moreover, a high normalized power density (P_max_/(ΔT)^2^) of 19.6 mW m^–2^ K^–2^ and a conversion efficiency of 1.34% (12.74% of the relative efficiency of Carnot) were obtained at a temperature difference of 35 K, which can be attributed the enhanced ion diffusion kinetics due to the addition of NH_4_^+^. Notably, the HTCCs can achieve continuous energy output for over 72 h when the temperature difference persists, demonstrating the promising potential of this hybrid ion co-insertion/extraction design in high-performance i-TEs for low-grade heat conversion and utilization.

## Experimental Section

### Synthesis of Porous HVO

The HVO was synthesized by an optimized template-assisted solvothermal method. Typically, 0.702 g of ammonium metavanadate (NH_4_VO_3_), 1.89 g of oxalic acid dihydrate (C_2_H_2_O_4_·2H_2_O), and 0.25 g of CTAB were dissolved into a mixed solvent of ethylene glycol (45 mL) and deionized water (15 mL). After stirring for about 30 min, the mixture was transferred into a stainless steel autoclave lined with Teflon and kept at a temperature of 180 °C for a full day. After cooling, the resulting mixture was washed repeatedly with ethanol and deionized water until the supernatant became clear and then dried at 80 °C overnight to obtain the product.

### Electrochemical Measurements

Thermoelectrochemical performance tests of HTCCs were conducted in a non-isothermal H-type cell on a standard electrochemical workstation (CHI760E). The temperature difference between the two chambers was controlled using a consistent temperature water bath. On the other hand, the three-electrode Swagelok cell arrangement was used for the half-cell tests. The Biologic VMP-300 workstation was used to record the electrochemical impedance spectroscopy (EIS) curves. The electrochemical workstation (CS310X, Wuhan Corrtest Instrument Corp., Ltd.) was used to record the galvanostatic charge/discharge (GCD) and the cyclic voltammetry (CV) curves. GITT, rate capability, and cyclic stability were measured using the CT3001A Land Battery Test System. To prepare the working electrode, the active material, conductive agent (acetylene black), and bonding agent (polyvinylidene fluoride) were mixed in a mass ratio of 7:2:1. This mixture was then disseminated in N-methyl-2-pyrrolidone (NMP) to form a slurry, and the slurry was painted on carbon fiber paper (CFP) current collectors with a diameter of 1.2 cm.

### Theoretical Calculations Methods

Density functional theory (DFT) calculations were conducted using the Vienna Ab Initio Simulation Package (VASP). The exchange correlation energy was described by the Perdew–Burke–Ernzerhof (PBE) functional within the generalized gradient approximation (GGA) and the projector augmented wave (PAW) method. The cutoff energy for the planewave basis was set to 530 eV and electron smearing with a width of 0.2 eV was adopted using Gaussian smearing technique. The k-point grid with a size of 3 × 3 × 3 was used to sample the Brillouin zone. The convergence criteria for geometry optimization are 10^–5^ eV and less than 0.03 eV Å^−1^, respectively. The van der Waals interactions were considered using the DFT-D3 method with Becke–Jonson damping. All calculations were performed using an optimized HVO unit cell (a = 9.27 Å, b = 9.52 Å, c = 8.01 Å) containing one structural water molecule. The Zn^2+^ and NH_4_^+^ ion migration barrier energies passing through V_2_O_5_·H_2_O were calculated by the climbing-image under elastic band (CI-NEB) method with force convergence set to be less than 0.05 eV Å^−1^.

## Results and Discussion

### Synthesis and Characterization of HVO

We have prepared hydrated vanadium pentoxide (HVO) material with nanoflower morphology using a template-assisted solvothermal method. The CTAB was added during the hydrothermal synthesis process as a template agent, which can effectively regulate the morphology of the product. As shown in Fig. S1a, b, the HVO sample displays a flower-like structure comprised of nanosheets, each approximately 500 nm in length and 20 nm in thickness. These nanosheets are stacked and self-assembled, forming a porous three-dimensional network. This layout significantly enhances the material's specific surface area, improving the wettability of the electrolyte and offering more paths for the diffusion of ions. The energy-dispersive X-ray spectrometry elements mapping images in Fig. S1c, d show a homogeneous distribution of vanadium and oxygen elements, affirming the structural integrity at the nanoscale. As shown in Fig. S2a, the N_2_ adsorption–desorption isotherm of HVO displays a characteristic type IV curve with a distinct hysteresis loop in the relative pressure range of 0.6 to 1.0, signifying the presence of mesopores. The steep rise in adsorption at elevated relative pressures (P/P₀ > 0.9) indicates the presence of larger macropores. The specific surface area of HVO was calculated to be 26.33 m^2^ g^–1^, and the large surface area facilitates the transport of electrolyte ions. Figure S2b illustrates the X-ray diffraction (XRD) pattern of the HVO sample. The diffraction peaks can be indexed with V_2_O_5_ (PDF#89-0612), V_2_O_5_·1.6H_2_O (PDF#40-1296), V_2_O_5_·0.5H_2_O (PDF#40-1297). The existence of low-angle peaks indicates a unique layered architecture, hence validating the effective synthesis of V_2_O_5_·xH_2_O. The Raman spectra (Fig. S2c) displays typical bands of V_2_O_5_ at 143, 198, 286, 406, 526, and 996 cm⁻^1^. The peak at 143 cm⁻^1^ is associated with V–O lattice bending, while the peaks at 198, 286, and 406 cm⁻^1^ are ascribed to V=O bond vibrations. The peak at 526 cm⁻^1^ is associated with V_3_-O stretching, whereas the peak at 996 cm⁻^1^ corresponds to V_2_-O stretching [37]. As depicted in Fig. S2d, the V 2*p* XPS spectrum indicates that vanadium in HVO exists predominantly in the + 5 oxidation state.

The thermogravimetric analysis curve exhibits a significant weight loss for HVO, where the weight loss below 100 °C is attributed to the removal of physically adsorbed water, and the weight loss between 100 and 330 °C is due to the removal of interlayer lattice water (Fig. S3). The curve stabilizes at temperatures above 400 °C, indicating the complete disappearance of water and structural transformation to anhydrous vanadium pentoxide. Further calculation reveals that the V_2_O_5_ phase in the HVO sample contained an average of ~ 1.1 interlayer lattice water molecules. The presence of interlayer lattice water molecules helps to improve the stability of the layered structure and mitigate the volume expansion during charging and discharging [38]. More importantly, they not only increase the interlayer spacing of HVO and widen the ion diffusion channels, but also participate in the diffusion process of NH_4_^+^, which greatly improves the diffusion rate of NH_4_^+^. This will be explained in detail in Sect. 3.5. As a verification, anhydrous vanadium pentoxide (VO) was prepared by removing the interlayer crystalline water in HVO (Fig. S4). As shown in Figs. S5 and S6, VO exhibits inferior performance compared to HVO in low-grade heat recovery and electrochemical energy storage, highlighting the crucial role of interlayer crystalline water.

### Evaluation of Thermoelectrochemical Performances

**Fig. 1:**
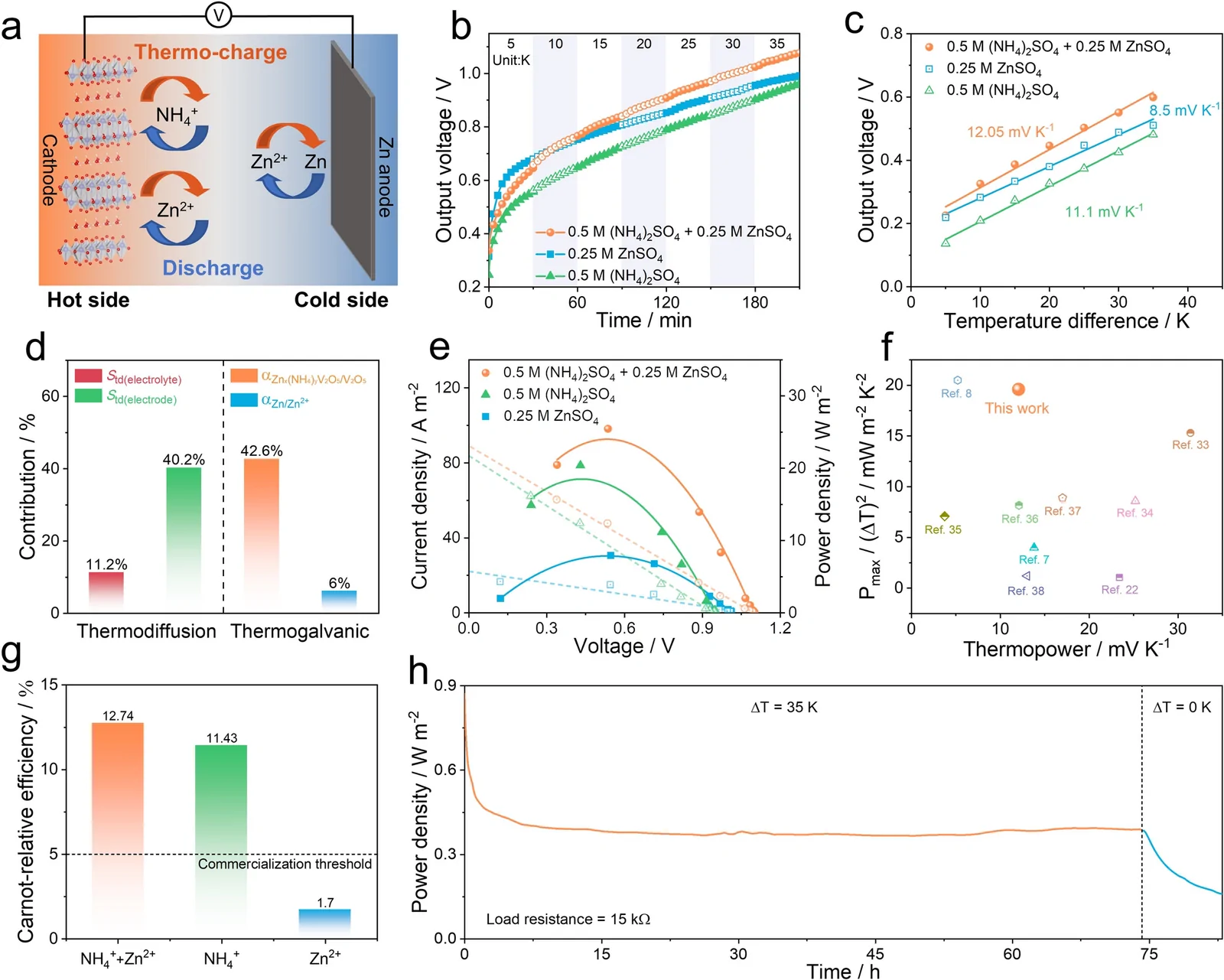
Thermoelectric performances of HTCCs.** a** Schematic illustration of HTCC. **b** Voltage variations at different temperature differences. **c** Thermopower fitting plots. **d** Contribution of various thermal processes to the total thermopower. **e** Plots of power and current densities under various load resistances. **f** Comparison in thermopower and normalized power density. **g** Carnot-relative efficiency of three thermal charging cells. **h** Long-term discharge of HTCC at 35 K temperature difference

To explore the differences in low-grade heat recovery capabilities of various cationic electrolytes, a series of tests were conducted using non-isothermal H-type electrolytic cells. Firstly, several electrolytes with different ratios were designed for this experiment. By screening them in terms of solubility, ionic conductivity, and electrochemical stability at room temperature (~ 25 °C), the 0.25 M ZnSO_4_ + 0.5 M (NH_4_)_2_SO_4_ solution was identified as the hybrid-ion electrolyte to conduct the subsequent experiments (optimization process is elaborated in Figs. S7–S9). For thermoelectrochemical performance tests, the HVO electrode was placed on the hot side as the cathode, and a Zn foil electrode was placed on the cold side as the anode. Additionally, three electrolyte systems including 0.5 M (NH_4_)_2_SO_4_ solution, 0.25 M ZnSO_4_ solution, and a hybrid solution of 0.5 M (NH_4_)_2_SO_4_ + 0.25 M ZnSO_4_ were comparatively studied. For ease of expression, they are abbreviated as ATCC (Ammonium-ion thermal charging cell), ZTCC (Zinc-ion thermal charging cell), and HTCC, respectively. As depicted in Fig. 1a, during discharge, Zn^2+^ is stripped from the Zn foil electrode, while NH_4_^+^ and Zn^2+^ are co-inserted into the HVO cathode. In the subsequent thermal charging process, the previously inserted cations in the cathode are gradually extracted and moved to the cold side with the application of the thermal field. Meantime, the diffused Zn^2+^ is plated on the Zn foil anode. The whole process realizes the conversion of low-grade heat into electrical energy and storage at the same time. Figure 1b records the voltage change of three thermal charging cells with heat input. The HTCC and ATCC exhibit similar curve shapes and a higher voltage growth rate compared to the ZTCC, which is attributed to the faster kinetic behavior of NH_4_^+^. On the other hand, the final output voltage of the HTCC and ZTCC is higher than that of ATCC due to the double electron transfer reaction of the Zn^2+^, with the output voltages of HTCC, ATCC, and ZTCC being 1.08, 0.96, and 0.99 V, respectively. HTCC combines the advantages of both Zn^2+^ and NH_4_^+^, achieving a higher output voltage and maintaining a faster voltage growth rate. To compare the thermoelectric conversion capabilities of the different systems quantitatively, we calculated the thermopower (${S}_{i}$) of the three thermal charging cells using Eq. 1:

1 $${S}_{i}=\frac{\Delta V}{\Delta T}$$

It is worth mentioning that we have removed the effect of self-charging in our calculations (Fig. S10). As plotted in Fig. 1c, the HTCC and ATCC have similar thermopower, which are 12.05 and 11.1 mV K^–1^, respectively. In contrast, the ZTCC has the lowest thermopower, only 8.5 mV K^–1^. As shown in Figs. 1d and S11, the high thermopower of HTCC is primarily attributed to the thermoextraction (${\alpha }_{{{Zn}_{x}({NH}_{4})}_{y}{V}_{2}{O}_{5}/{V}_{2}{O}_{5}}$) and thermodiffusion (${S}_{td(electrode)}$) processes of ions within the HVO material, which account for 42.6% and 40.2%, respectively. Additionally, the thermodiffusion of ions in the electrolyte (${S}_{td(electrolyte)}$) accounts for 11.2% and the plating/stripping process of Zn^2+^ on the Zn foil anode (${\alpha }_{Zn/{Zn}^{2+}}$) contribute 6%.

Energy output capacity is one of the most important indicators of a thermoelectric system. Here we use a series of fixed resistors (100 kΩ ~ 50 Ω) at a temperature difference of 35 K to study the performance of three thermal charging cells in terms of power density and current response. As shown in Fig. 1e, the maximum power densities of HTCC, ATCC, and ZTCC are 24, 18.5, and 7.9 W m^–2^, respectively. This represents a significant enhancement, where the power density of HTCC is approximately 204% higher than that of ZTCC and about 30% higher than that of ATCC. The fitted parabola of HTCC is very similar to that of ATCC, and both exhibit high power densities. This suggests that HTCC has successfully retained the faster kinetic behavior of NH_4_^+^. Additionally, the power density of HTCC is higher than that of ATCC, which can be attributed to the improved device's voltage resulting from the utilization of the Zn anode. In contrast, while ZTCC also has a relatively high voltage, its slower ion diffusion rate leads to a lower power density. Moreover, the normalized power density provides a more precise metric for comparing the thermoelectric conversion capabilities of different systems. The normalized power density of HTCC reaches 19.6 mW m^–2^ K^–2^, which is among the highest reported values for i-TEs (Fig. 1f) [7, 11, 15, 22, 39–43]. In addition, we compared the current responses of the three thermal charging cells. The current responses of the HTCC (90 A m^–2^) and ATCC (84 A m^–2^) were much higher than that of the ZTCC (22.5 A m^–2^). Further calculations revealed that the internal resistances (${R}_{cell}$) of HTCC, ATCC, and ZTCC were 106.9, 101.1, and 398 Ω, respectively (Fig. S12). The energy conversion efficiency (η) and Carnot-relative efficiency (${\eta }_{r}$) of a device are also crucial metrics for assessing thermoelectric conversion capabilities. Here, we calculated the η using Eq. 2 [21, 40, 44]:

2 $$\eta =\frac{{{S}_{i}}^{2}\Delta T}{4\kappa }\bullet \frac{d}{A{R}_{cell}}$$

where κ is the thermal conductivity, d is the inter-electrode spacing, A is the cross sectional area. As shown in Fig. S13, the η of HTCC, ATCC, and ZTCC are 1.34%, 1.2%, and 0.18%, respectively (Details of efficiency calculation are shown in Table S1.). The ${\eta }_{r}$ was further calculated using Eq. 3 [15]:

3 $${\eta }_{r}=\frac{\eta }{\Delta T/{T}_{H}}$$

where ΔT is the temperature difference between the hot and cold sides and ${T}_{H}$ is the hot side temperature. As shown in Fig. 1g, the ${\eta }_{r}$ of HTCC, ATCC, and ZTCC are 12.74%, 11.43%, and 1.7%, respectively. The high ${\eta }_{r}$ of HTCC has exceeded the predicted commercialization threshold and most of the previously reported works, demonstrating high potential for practical applications.

The continuous energy output capability of thermal charging cells is also important. Figure S14 shows the power density variation curves of three thermal charging cells during long-term discharge under a temperature difference of 35 K. For HTCC (Fig. S14a), the power density experiences a sharp drop when initially loaded with a resistance of 2 kΩ, but it stabilizes after approximately 500 s. In contrast, ATCC and ZTCC fail to reach a stable state when loaded with the same resistance, resulting in a continuous decline in power density. The energy densities (E) of the three thermal charging cells are further calculated using Eq. 4 [42]:

4 E=∫Pdt

In this study, the output power was integrated over a period of 1 h to determine the energy density of the thermal charging cells. When loaded with a 2 kΩ resistance, all three thermal charging cells achieved their maximum energy density. Specifically, the ${E}_{1h}$ of HTCC, ATCC, and ZTCC are calculated to be 4.5, 3.8, and 4 kJ m^–2^, respectively. In practical applications such as wearable devices or IoTs sensors, there is often a need to continuously power electrical devices under a certain temperature difference. To this end, we conducted continuous discharge of HTCC with a resistance of 15 kΩ at a temperature difference of 35 K, while simultaneously monitoring changes in power density. As shown in Fig. 1h, the power density gradually stabilizes after about 5 h and can be maintained for over 72 h. At this time, the energy input from the thermal charging process and the energy output from discharging reach a balance, achieving continuous conversion and release of thermal energy. When the temperature difference across the HTCC was eliminated, the output power showed a significant decrease, thereby verifying that the temperature difference plays a key role in the aforementioned energy conversion process. Figures S15-S17 demonstrate that both the HVO cathode material and Zn anode maintain stability after prolonged operation. Additionally, Fig. S18 shows that HTCC can achieve extended continuous operation even at higher power densities.

### Mechanism Analysis of Zn^2+^/NH_4_^+^ Co-insertion and Thermoextraction

**Fig. 2:**
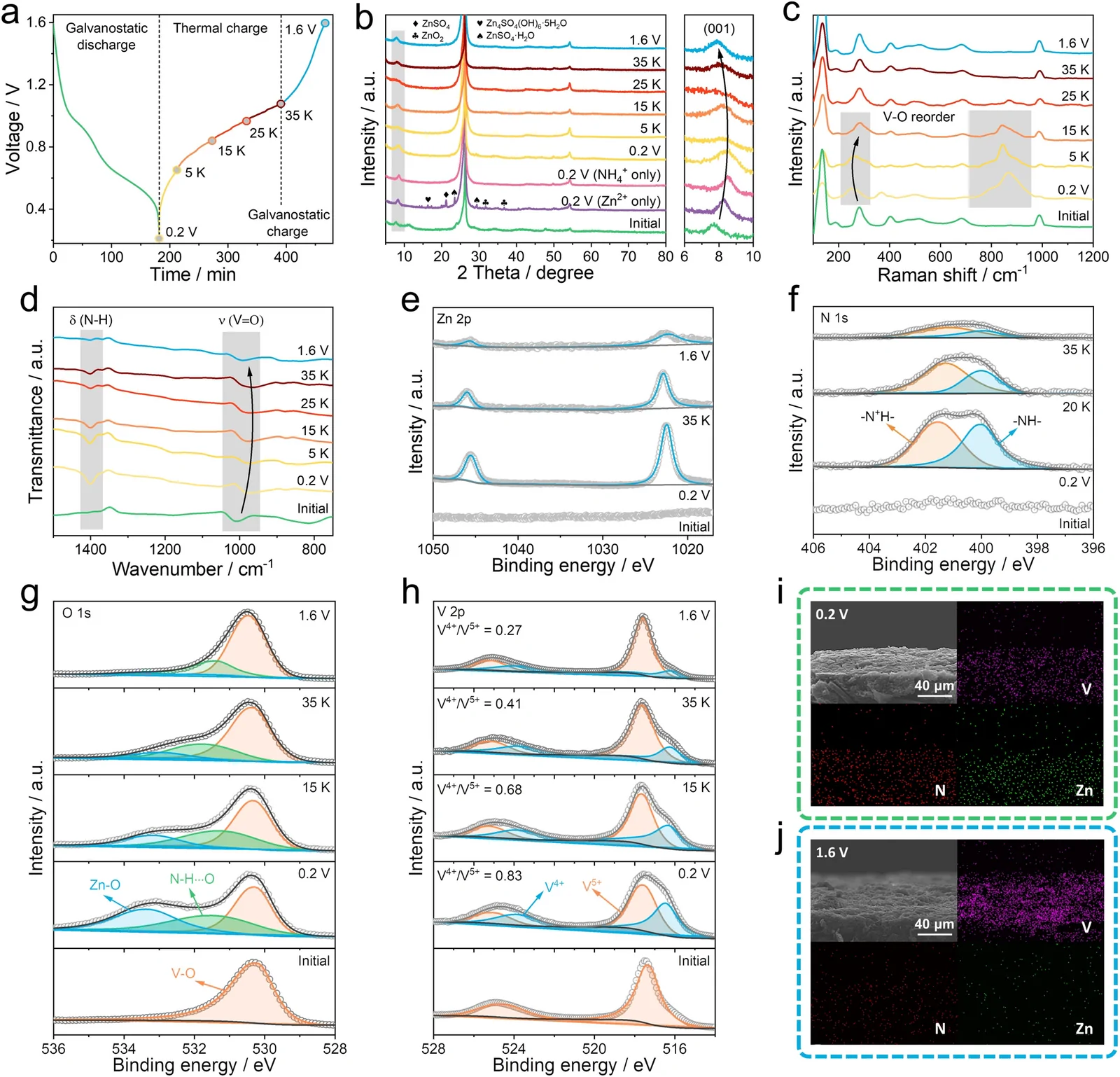
Mechanism analysis of HTCCs. **a** Voltage change curve during different processes in hybrid electrolytes with marked points for ex situ investigations. Ex situ **b** XRD patterns, **c** Raman spectra, **d** FTIR spectra, **e–h** Zn 2*p*, N 1*s*, O 1*s*, and V 2*p* XPS spectra, and **i, j** SEM–EDS elemental mapping images of the HVO electrodes at selected states

To gain insight into the energy conversion and storage mechanisms of HVO in hybrid electrolytes, a series of ex situ characterizations were conducted to monitor the cathode during representative stages as indicated in Fig. 2a: fully discharged state (0.2 V), thermal charging states (5–35 K), and fully charged state (1.6 V) to determine the maximum ion storage capacity and evaluate the structural reversibility of the entire process. Firstly, Fig. 2b presents the XRD patterns of the electrode in these different states. All XRD patterns show distinct peaks at approximately 26.4° and 54.4°, which are characteristic peaks of the carbon fiber current collector. A key question that needs to be clarified is whether Zn^2+^ and NH_4_^+^ are indeed co-inserted into the interlayers of the HVO cathode material. To address this, we additionally collected XRD patterns of the HVO cathode in the fully discharged state in 0.25 M ZnSO_4_ electrolyte (0.2 V, Zn^2+^ only) and 0.5 M (NH_4_)_2_SO_4_ electrolyte (0.2 V, NH_4_^+^ only). The XRD patterns in all three electrolytes exhibit a clear shift of the (001) plane to a higher angle during discharging, which is attributed to the contraction of the layer spacing due to the electrostatic interactions between Zn^2+^/NH_4_^+^ and O^2−^ of V_2_O_5_ framework [29, 45–48]. Specifically, when the Zn^2+^ is inserted, the (001) plane shifts from 7.72° degrees to 8.26°, representing a reduction of the layer spacing from 11.41 to 10.66 Å. When the NH_4_^+^ is inserted, the layer spacing of (001) is further reduced to 10.43 Å. This is attributed to the stronger interaction force of the NH_4_^+^ due to the hydrogen bonding they form between the V_2_O_5_ layers [27]. It is noteworthy that in the 0.25 M ZnSO_4_ electrolyte, there is a severe issue with by-products and electrolyte residue on the electrode surface: Zn_4_SO_4_(OH)_6_·5H_2_O (JCPDS#00-039-0688), ZnO_2_ (JCPDS#01-076-1364), ZnSO_4_ (JCPDS#01-070-1255), and ZnSO_4_·H_2_O (JCPDS#00-001-0621). In contrast, in the 0.5 M (NH_4_)_2_SO_4_ electrolyte and the hybrid electrolyte, the issue of by-products is minimal [49]. During the subsequent thermal charging process, the (001) plane gradually returns to a lower angle, indicating that Zn^2+^ and NH_4_^+^ are thermoextracted from the crystal lattice. Moreover, the (001) plane returned very close to its original position (7.88°) after galvanostatic charging, further indicating that the insertion and extraction of Zn^2+^ and NH_4_^+^ are reversible processes [22]. Notably, the slight deviation of the (001) plane from its original position suggests that traces of inserted Zn^2+^ and NH_4_^+^ remain in the HVO interlayer, acting as “interlayer pillars” to enhance the electrode structure [26]. Figure 2c summarizes the Raman spectra under different states. The initial state electrode showed Raman characteristic peaks similar to those of the HVO material, with the typical D_2h_ point symmetry of the V–O layer. When discharged to 0.2 V, a distinct new peak appeared near 867 cm⁻^1^, while the V=O signal shifted from 280 to 250 cm⁻^1^. This indicates that the co-insertion of Zn^2+^/NH_4_^+^ leads to the reordering of the V–O layers. It is worth noting that during the charging process, the Raman spectrum gradually reverts to the original D_2h_ characteristics, further confirming the high reversibility of the HVO electrode [26, 50, 51].

The reason why NH_4_^+^ has faster kinetic behavior compared to other ions was also systematically investigated in this study. The ex situ FTIR spectra can effectively analyze the interaction between NH_4_^+^ and HVO electrode materials during the insertion/extraction processes. As shown in Figs. 2d and S19, when discharged to 0.2 V, the new peaks located at 1401 and 3117 cm⁻^1^ can be ascribed to the bending and stretching vibrations of N–H, which strongly proves the insertion of NH_4_^+^. Furthermore, the new peak that appears at 3031 cm⁻^1^ represents the formation of N–H···O hydrogen bonds between the inserted NH₄⁺ and the V–O layers, which notably enhances the ion diffusion kinetics [29, 47]. Additionally, the peak corresponding to the stretching of the V=O bond shifted from around 1014 to 977 cm⁻^1^, indicating that during the ion insertion process, some V^5+^ in HVO was partially reduced to V^4+^ [52]. In the subsequent charging process, these phenomena gradually reverted.

The ex situ Zn 2*p* and N 1*s* XPS spectra depicted in Fig. 2e, f show clear Zn^2+^ and NH₄⁺ signals in the fully discharged state. Moreover, as illustrated in Fig. 2g, the O 1*s* XPS spectrum in the fully discharged state also exhibits distinct Zn–O signals (~ 533.4 eV) and N–H···O signals (~ 531.4 eV), further confirming the co-insertion of Zn^2+^ and NH_4_^+^. The appearance of the N–H···O signal also reaffirms the hydrogen bond interaction formed between the NH_4_^+^ and the V–O layers [47, 53, 54]. When the voltage is increased to 1.6 V, the signals mentioned above gradually disappear. The valence evolution and electronic interactions of the HVO cathode were revealed by ex situ V 2*p* XPS spectra in Fig. 2h. When discharged to 0.2 V, the proportion of V^4+^ significantly increased, and after charging to 1.6 V, the vanadium valence state almost recovered to its initial state, which is consistent with the results of the ex situ FTIR spectra. Finally, the SEM–EDS mapping images of the HVO electrode in charged and discharged states further demonstrated the co-insertion/extraction mechanism of Zn^2+^ and NH_4_^+^ (Fig. 2i, j).

### Electrochemical Characterizations

**Fig. 3:**
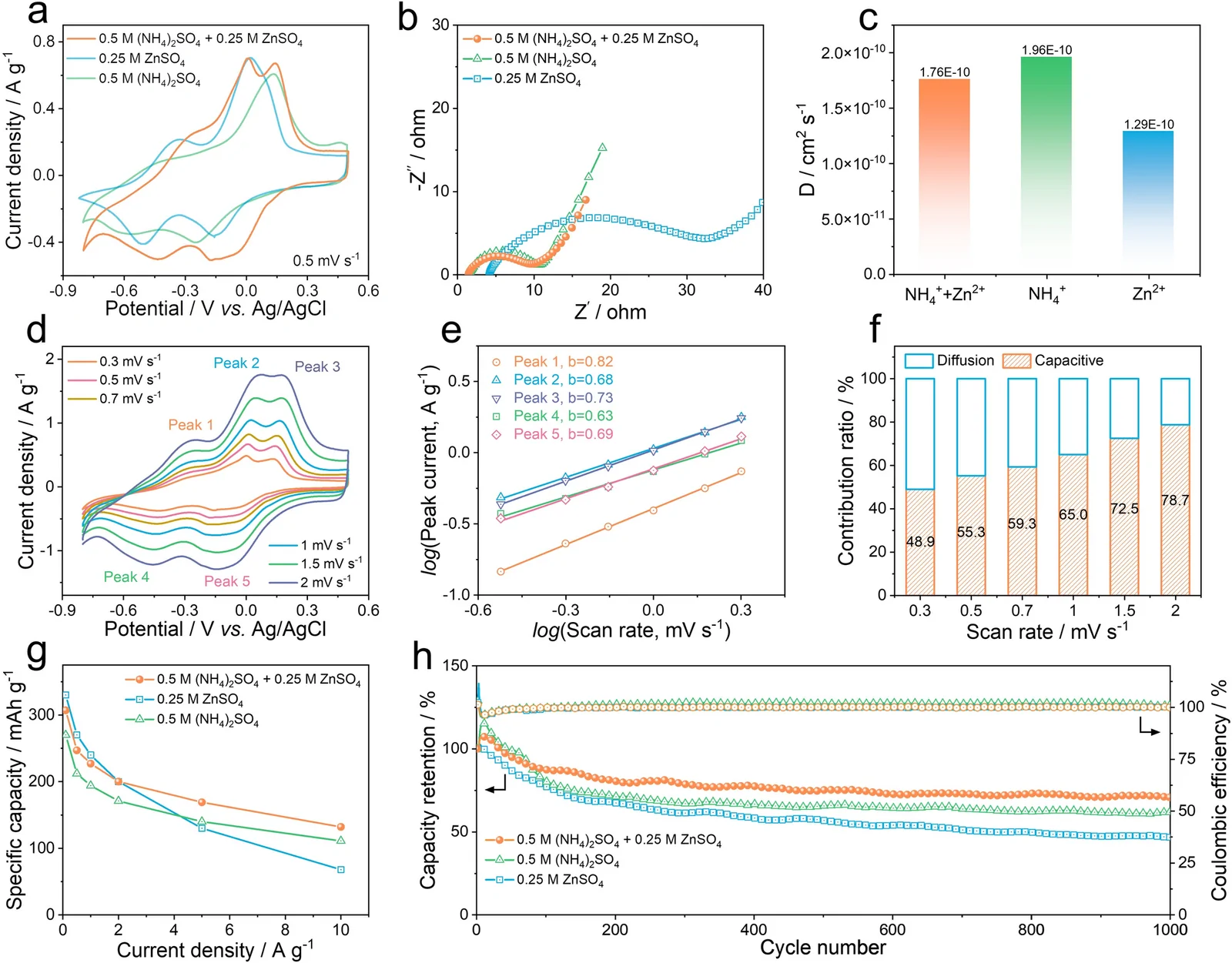
Electrochemical characterization and kinetic analysis of HVO electrode in hybrid electrolyte. **a** CV curves of HVO cathode in different electrolytes at 0.5 mV s^–1^. **b** EIS spectra of different electrolyte cells. **c** Ion diffusion coefficient obtained by GITT curves in different electrolytes. **d** CV curves of HVO cathode in hybrid electrolyte at different scan rates from 0.3 to 2.0 mV s^−1^. **e** log(i) versus log(v) plots at redox peaks. **f** The contribution percentage of diffusion-controlled capacities and capacitive capacities. **g** Comparison of rate properties of HVO cathode in different electrolytes. **h** Long-term cycle life of HVO cathode in different electrolytes at 2 A g^–1^

To further clarify the migration and storage behaviors of Zn^2+^ and NH_4_^+^ within the cathode material, a series of three-electrode cell tests were conducted. As shown in Fig. 3a, the CV curve of the HVO in Zn^2+^ aqueous electrolyte shows two distinct pairs of peaks at around 0.01/–0.0.15 and –0.39/–0.51 V, which can be attributed to a two-step reaction associated with Zn^2+^ insertion/extraction. In the hybrid electrolyte, the CV curve maintained that shape as a whole and a new oxidation peak appeared at ~ 0.14 V, similar to that observed in the NH_4_^+^ electrolyte. It proved that NH_4_^+^ was involved in the electrochemical reaction process. In addition, the hybrid electrolyte has a pH of 4.17 and is weakly acidic. We prepared a dilute H_2_SO_4_ solution also with a pH of 4.17 and performed CV tests under the same conditions to investigate whether hydrogen ions or hydrated hydrogen ions in the electrolyte were involved in the reaction. As shown in Fig. S20, the current response of HVO in dilute H_2_SO_4_ is very small, and by integrating the two CV curves, it is found that the capacity of the HVO electrode in dilute H_2_SO_4_ solution is only about 5% of that in the hybrid electrolyte. In conclusion, it can be determined that the main ions participating in the reaction in the hybrid electrolyte are Zn^2+^ and NH_4_^+^.

To further investigate the kinetics of HVO electrodes for the storage of various ions, we carried out a series of kinetics analyses. To begin with, the three cells exhibit similar EIS curve shapes (Fig. 3b), indicating that they have similar ion transport paths. In the high-frequency region, the intersection points of the EIS plot with the Z' axis corresponds to the contact impedance. The maximal contact resistance obtained by the Zn^2^⁺ based cell (3.93 Ω) suggests stronger ionic polarization effects compared to NH₄⁺-containing systems, consistent with the larger Stokes radius and higher charge density of hydrated Zn^2^⁺.In addition, the charge transfer resistance (R_ct_) for the Zn^2+^/NH_4_^+^, NH_4_^+^, and Zn^2+^ based cells are 8.62, 6.98, and 22.04 Ω. It is evident that the incorporation of NH₄⁺ significantly enhances the ionic reaction rate of the hybrid electrolyte. The galvanostatic intermittent titration technique (GITT) was also utilized to analyze the ionic diffusion coefficients. As depicted in Fig. 3c, the average ionic diffusion coefficient of the hybrid electrolyte cell can be calculated to be 1.76 × 10^–10^ cm^2^ s^–1^, which is significantly higher than 1.29 × 10^–10^ cm^2^ s^–1^ for the Zn^2^⁺ based cell (for detailed calculations, see Fig. S21). Additionally, as shown in Fig. 3d, we performed CV tests on hybrid electrolyte cell with scan rates ranging from 0.3 to 2 mV s^−1^. With the increase in scan rate, the CV curves maintain similar shapes, indicating good electrochemical reversibility. To enhance comprehension of the charge storage kinetics in the HVO host, we quantitatively separated the diffusion-controlled and capacitive contributions to the recorded current. The correlation between the recorded peak current (i) and scan rate (v) in a CV scan can be described by Eq. 5:

5 $$i=a{v}^{b}$$

A b value of 0.5 signifies that the current is controlled by semi-infinite diffusion, while b =1 denotes capacitive behavior. For HVO in the hybrid electrolyte, the *b*-values of the five peaks are 0.82, 0.68, 0.73, 0.63, and 0.69, respectively (Fig. 3e). This means that the charge storage process is controlled by both diffusion and capacitance processes. Additionally, the capacity may be precisely separated into two components: capacitance (${k}_{1}v$) and diffusion-controlled (${k}_{2}{v}^{1/2}$), as determined by Eq. 6. Figure 3f shows that as the scan rate increases, the capacitive contribution ratio steadily rises from 48.9% to 78.7%.

6 $$i={i}_{\mathrm{cap}}+{i}_{\mathrm{diff}}={k}_{1}v+{k}_{2}{v}^{1/2}$$

The storage capacity of the HVO cathode for different ions was compared through galvanostatic charge/discharge (GCD) experiments (Figs. 3g and S22). Due to the two-electron reaction of Zn^2+^, HVO in the Zn^2+^ electrolyte shows the highest specific capacity at low current densities. However, the HVO cathode exhibits rapid capacity fading at increasing current densities in the Zn^2^⁺ electrolyte, while demonstrating markedly superior rate capability for NH₄⁺ storage. This is due to the smaller hydrated ion size (3.31 Å) and unique topological chemistry of NH_4_^+^, which results in a faster diffusion rate in the electrolyte and electrode materials. It is noteworthy that the hybrid electrolyte simultaneously achieves both high capacity and excellent rate capability. Moreover, the HVO cathode in hybrid electrolyte demonstrated superior capacity retention, achieving 71% retention after 1,000 GCD cycles, significantly outperforming the other two electrolytes (Fig. 3h). The significantly improved cycling stability is further supported by Zn||Zn symmetric cell tests, which demonstrate a lower polarization voltage in the hybrid electrolyte, indicating that the presence of NH₄⁺ ions promote more uniform Zn deposition (Fig. S23). The above analysis indicates that NH_4_^+^ has more rapid kinetic characteristics compared to Zn^2+^, which is crucial for enhancing the energy conversion capability.

### Density Functional Theory Calculations

**Fig. 4:**
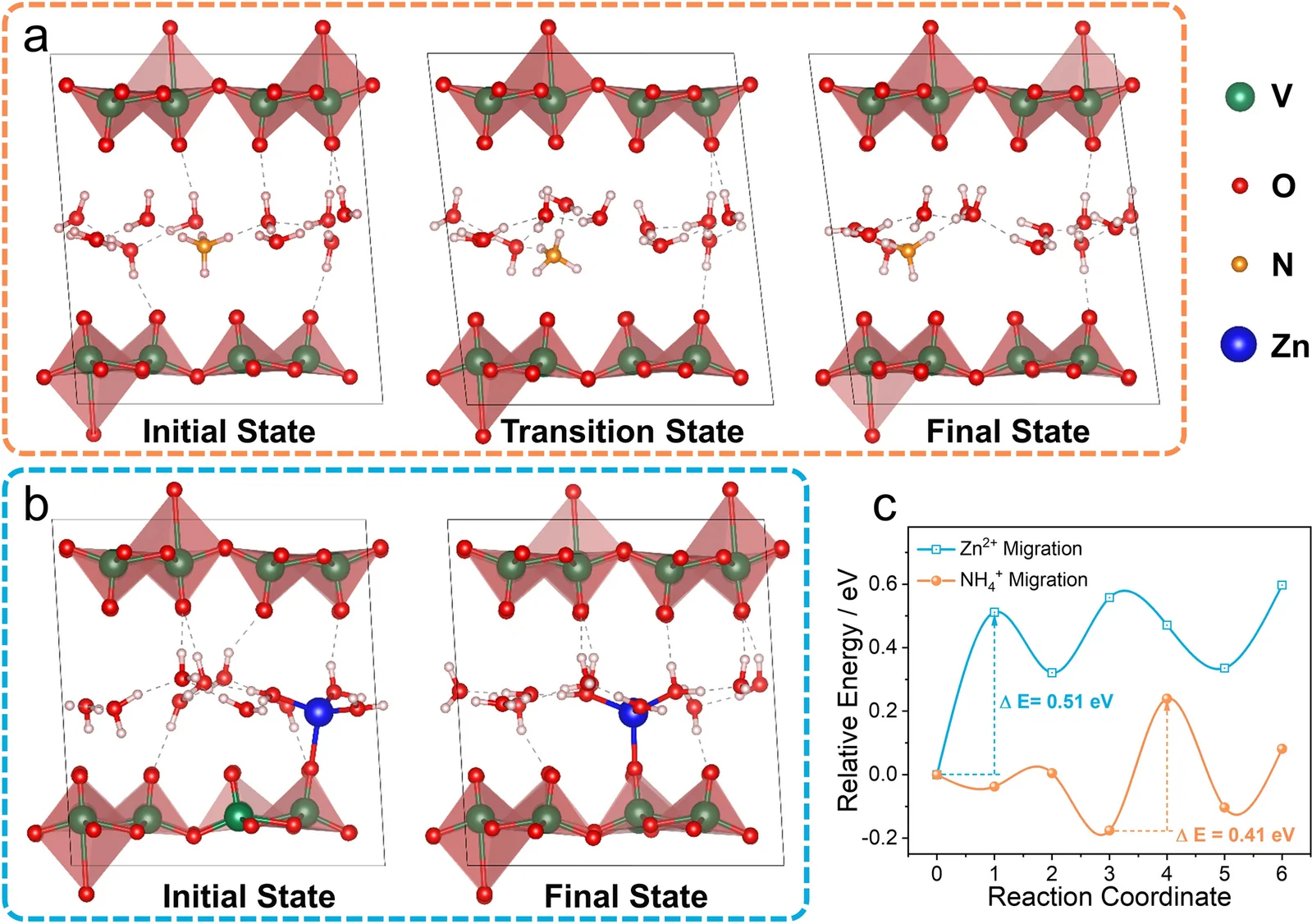
Simulation of ion migration processes. The evolution of different states during the ion diffusion process: **a** NH_4_^+^, **b** Zn^2+^. **c** Diffusion energy barrier of NH_4_^+^ and Zn^2+^ in V_2_O_5_·H_2_O

The interactions between NH_4_^+^ and Zn^2+^ with V_2_O_5_·H_2_O are also investigated through density functional theory (DFT) calculations. The simulation result of the NH_4_^+^ migration process is shown in Fig. 4a. It can be observed that during the diffusion process, NH_4_^+^ primarily relies on the hydrogen bonding interactions formed with the interlayer crystalline water of V_2_O_5_·H_2_O to achieve migration. Specifically, in the initial state, NH_4_^+^ inserted into V_2_O_5_·H_2_O forms three hydrogen bonds with the interlayer crystalline water. During the diffusion process, NH_4_^+^ breaks a hydrogen bond by twisting and rotating, and subsequently forms a new hydrogen bond in the forward direction. At the transition state, only two hydrogen bonds connect NH₄⁺ to the interlayer water. In the final state, three hydrogen bonds are reformed, completing the diffusion process. According to the experimental and DFT simulation results, the diffusion behavior of NH_4_^+^ in HVO materials is in accordance with the “monkey bars swinging” model previously proposed by Dong et al., but with a slight difference [29]. The previous model believed that NH_4_^+^ mainly relied on hydrogen bonding interactions with the V_2_O_5_ framework to achieve rapid diffusion. However, in this work, the prepared HVO material contained more crystalline water. The interlayer water not only widened the interlayer spacing and stabilized the structure as "interlayer pillars," but also actively participated in the NH_4_^+^ diffusion process, providing a more efficient pathway for its migration. This unique diffusion mechanism is the primary reason for the superior kinetic behavior of NH_4_^+^. Figure 4b illustrates the diffusion process of Zn^2+^ between the layers of V_2_O_5_·H_2_O. During the diffusion process, Zn^2+^ interacts with both the interlayer crystalline water and the V_2_O_5_ framework, forming stronger ionic bonds. Moreover, the spherical Zn^2^⁺ lacks a specific orientation preference, and the disruption of ionic bonds during its diffusion demands higher energy input, leading to a slower migration rate. This kinetic limitation is further highlighted when compared to NH₄⁺, whose lighter mass and smaller hydrated radius significantly promote its diffusion through the interlayers relative to Zn^2^⁺. The ease of diffusion for different ions in V_2_O_5_·H_2_O was quantitatively assessed by comparing their energy barriers. As shown in Fig. 4c, the diffusion barriers for NH₄⁺ and Zn^2^⁺ are 0.41 and 0.51 eV, respectively. The lower barrier of NH₄⁺ corroborates its ability to migrate more rapidly within the HVO material.

### Demonstration of Wearable Devices

**Fig. 5:**
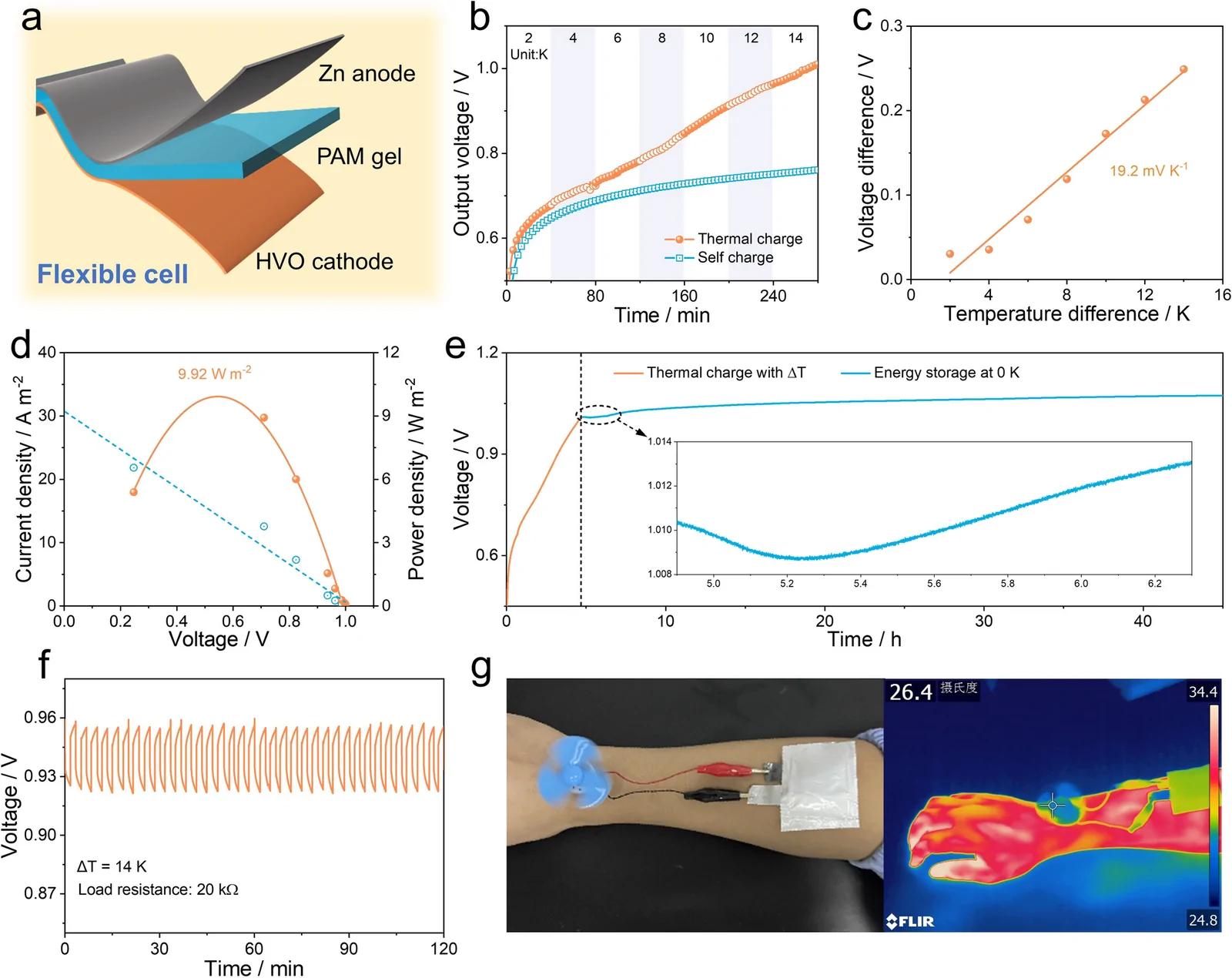
Construction and performance of wearable quasi-solid-state HTCCs. **a** Schematic configuration of HVO-based quasi-solid-state HTCCs. **b** Voltage variation curves with and without temperature difference. **c** Thermopower fitting plot. **d** Plots of power and current densities under various load resistances. **e** Voltage holding curve after removing temperature differences. **f** Thermal stability with loading resistance of 20 kΩ. **g** A small fan driven by one as-fabricated quasi-solid-state HTCC

To meet practical application requirements, safe, flexible, and cost-effective quasi-solid-state HTCCs were assembled using polyacrylamide (PAM) hydrogel electrolyte (Figs. 5a and S24-S25). Figure 5b illustrates the voltage variation over time with and without an applied temperature difference. It is evident that after applying a temperature difference, the quasi-solid-state HTCC exhibits a completely different voltage growth behavior. After subtracting the self-charging contribution, the pure thermally induced voltage at a temperature difference of 14 K is 249.1 mV, and the thermopower is calculated to be 19.2 mV K^−1^ (Fig. 5c). Figure 5d illustrates the relationship between current density, power density, and the corresponding voltage. The quasi-solid-state HTCC achieves a maximum power density of 9.92 W m^−2^ around 0.54 V. Additionally, the device obtains a short-circuit current density of 30.7 A m^−2^, demonstrating its excellent energy output capability. Meanwhile, to substantiate the capability of HTCCs for the integration of thermoelectric conversion and energy storage, we documented the voltage response completing the thermal charging process and eliminating the temperature difference. As shown in Fig. 5e, upon the temperature difference is removed, the quasi-solid-state HTCC experiences a slight voltage drop. This phenomenon may be due to the momentary disordering of ions in the electrolyte, which were originally orderly, resulting in a decrease in the ion concentration difference between the cold and hot sides. However, it is encouraging that after this brief voltage drop, the voltage output of the HTCC can quickly stabilize and maintain this for an extended period. In addition, the hybrid ion system also has good energy storage capability (Fig. S26). Figure 5f shows that the quasi-solid-state HTCC has a good capability of repeated thermal charging and discharging, and can work stably for more than 30 cycles. In an environment of 26.4 °C, we placed the hot electrode side of the quasi-solid-state HTCC directly onto the bare skin of an arm, with the other side exposed to the air. The device was capable of driving a small fan to operate continuously (Fig. 5g). The infrared thermal image shows a temperature difference of only 5.7 °C between the hot and the cold side.

## Conclusion

In summary, this work demonstrates the successful application of the Zn^2+^/NH_4_^+^ hybrid ion system in the field of thermal charging cells. Utilizing the Zn^2+^/NH_4_^+^ co-insertion/thermoextraction mechanism, the HTCCs effectively combine the high-voltage advantage of the two-electron reaction of Zn^2+^ and the low standard electrode potential of Zn metal anode, with the rapid kinetic behavior of NH_4_^+^. This enables the HTCCs to achieve a thermopower of 12.05 mV K^−1^, a power density of 24 W m^−2^, a normalized power density of 19.6 mW m^−2^ K^−2^, and a high relative Carnot efficiency of 12.74% under a 35 K temperature difference. It is worth mentioning that under a sustained temperature difference, the HTCCs can operate continuously for over 72 h, providing the possibility for long-term self-powered devices. Furthermore, DFT calculations reveal that NH_4_^+^ primarily interacts with interlayer crystalline water in V_2_O_5_·H_2_O cathode materials through hydrogen bonding during diffusion, which contributes to its rapid migration rate. Additionally, wearable devices prepared with gel electrolytes can utilize body temperature as a heat source to power small electronic devices, showing a promising application prospect. Compared with other design strategies for thermal charging cells, this study provides a simple and efficient method to improve the energy conversion capability and practicality.

## Supplementary Information

Below is the link to the electronic supplementary material.

Supplementary file1 (DOCX 5568 KB)
