# Supplementary material for: A High-Performance Thermal Charging Cell with High Power Density and Long Runtime Enabled by Zn2+ and NH4+ Co-insertion
Source: Nanomicro Lett. 2026 Jan 5;18:173. doi: 10.1007/s40820-025-02011-9 (PMC12765772; doi:10.1007/s40820-025-02011-9)
Supplement: Supplementary file 1 — Supplementary file1 (DOCX 5568 KB) [file 40820_2025_2011_MOESM1_ESM.docx]

Supporting Information for

**A High-Performance Thermal Charging Cell with High Power Density and Long Runtime Enabled by Zn^2+^ and NH^4+^ Co-Insertion**

Zhiwei Han^1#^, Shengliang Zhang^1#^*, Helang Huang^1^, Jing Wang^1^, Hui Dou^1^, Tianran Zhang^2^, Xiaogang Zhang^1^*

^1^ Jiangsu Key Laboratory of Electrochemical Energy Storage Technologies, College of Material Science and Technology, Nanjing University of Aeronautics and Astronautics, Nanjing 211106, P. R. China

^2^ College of Material Science and Opto-Electronic Technology, University of Chinese Academy of Sciences, Beijing 100049, P. R. China

^#^ Zhiwei Han and Shengliang Zhang contribute equally to this work.

*Corresponding authors. E-mail: [zhangsl@nuaa.edu.cn](mailto:zhangsl@nuaa.edu.cn) (Shengliang Zhang); [azhangxg@nuaa.edu.cn](mailto:azhangxg@nuaa.edu.cn) (Xiaogang Zhang)

**S1 Experimental Section**

**S1.1 Preparation of quasi-solid-state hydrogel electrolyte**

The hydrogel was prepared by dispersing 2 g acrylamide (AM), 1.2 mg N,N′-methylenebisacrylamide (MBA) and 8 μL N,N,N′,N′-Tetramethylethylenediamine (TMEDA) in an ice bath to deionized water in turn, and finally adding 0.18 g potassium persulfate (K_2_S_2_O_8_), stirring violently, and polymerizing under ultraviolet lamp (60 W, 365 nm) for 25 minutes. Finally, a transparent and flexible PAM gel was prepared by soaking in a hybrid solution of 0.25 M ZnSO₄ and 0.5 M (NH₄)₂SO₄.

**S1.2 Materials Characterizations**

The morphology of the as-obtained materials was observed by scanning electron microscope measurement (SEM, Scios 2 HiVac QUANTAX 200 with XFlash®6|60). X-ray diffraction (XRD) patterns were recorded from a PANaly-tical Empyrean diffractometer with Cu Kα radiation (λ = 1.5406Å). The surface chemical states of samples were detected by X-ray photo-electron spectrometer (XPS, Thermo Scientific K-Alpha). The Raman spectra were collected by the Horiba Scientific LabRAM HR with an excitation wavelength of 532 nm. Thermogravimetric analysis (TGA) curve was recorded on aMettler-Toledo STARe SW15.00 analyzer with a heating rate of 10 ^o^C min^−1^ under air flow. Fourier transform-infrared spectrum was tested on an FT-IR spectrophotometer (Bruker INVENIO-R). The specific surface area was studied by ASAP 2460 analyzer (Micromeritics), and was analyzed by Brunauer-Emmett-Teller (BET) method.

**S2 Results and Discussion**


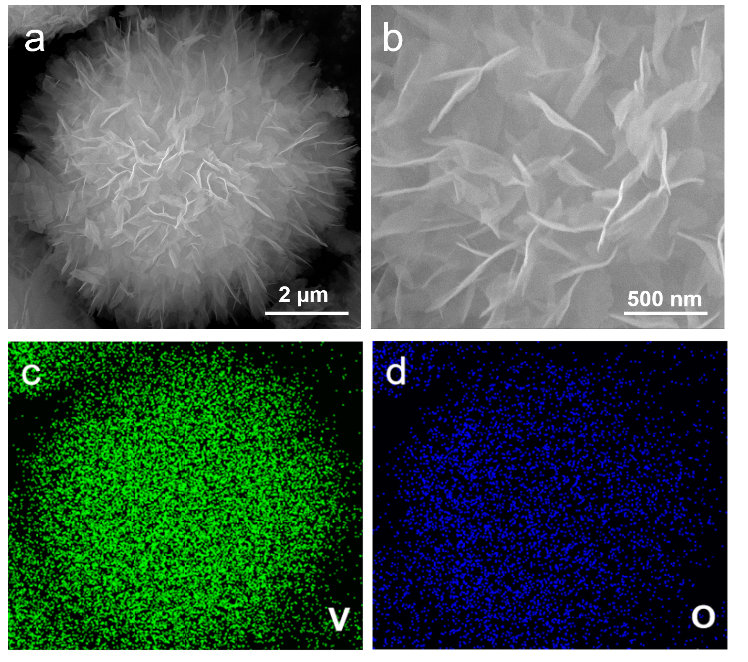


**Fig. S1** (**a, b**) SEM images of HVO. (**c, d**) EDX mapping images of HVO


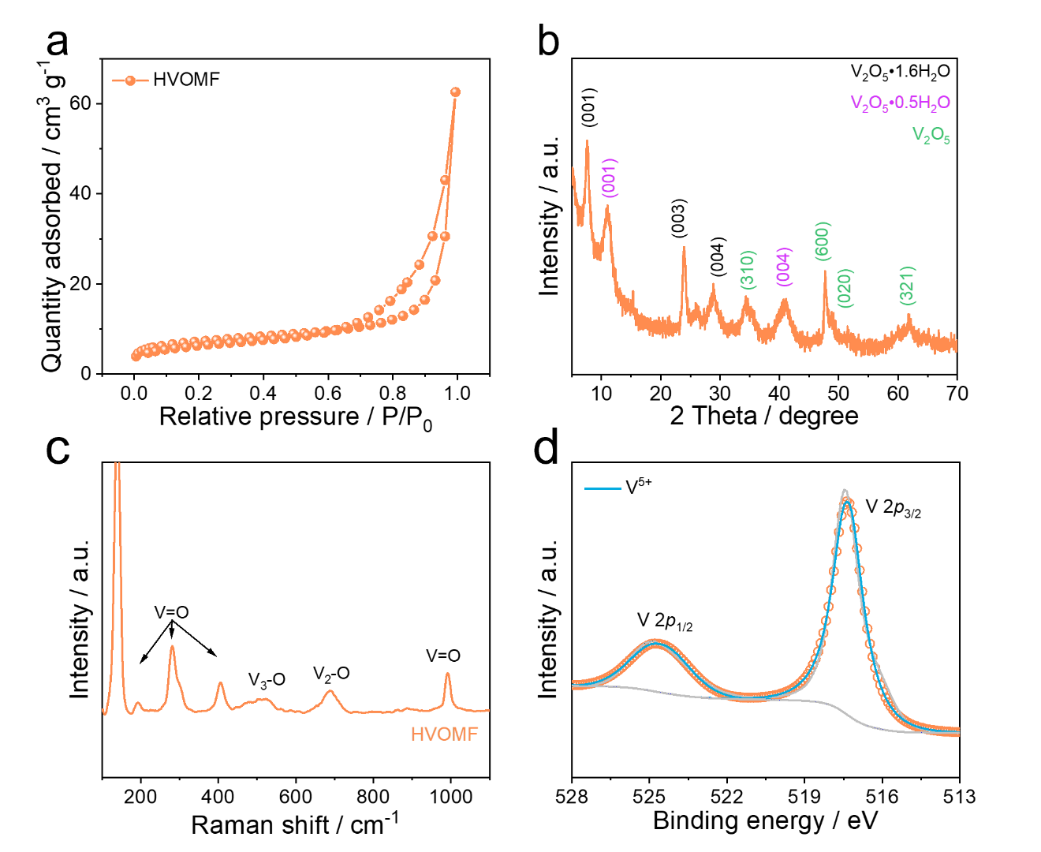


**Fig. S2** Characterization of HVO: (**a**) N_2_ adsorption-desorption isotherm. (**b**) XRD pattern. (**c**) Raman spectrum. (**d**) V 2p XPS spectra


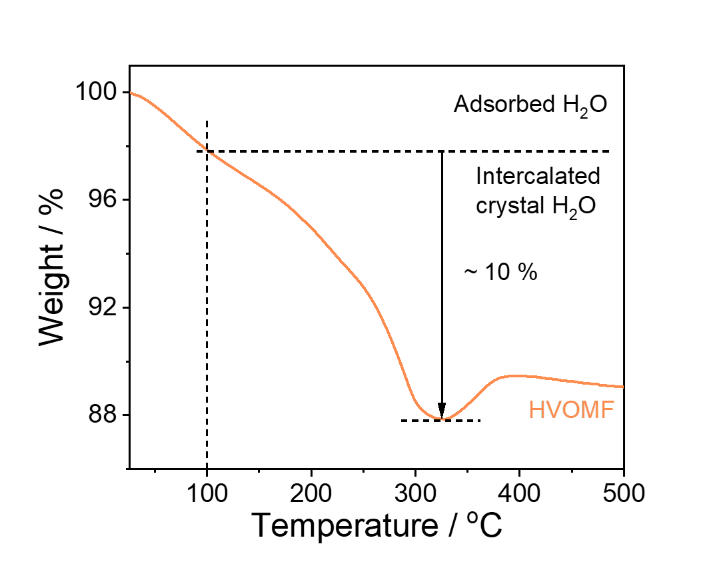


**Fig. S3** TGA curve of HVO
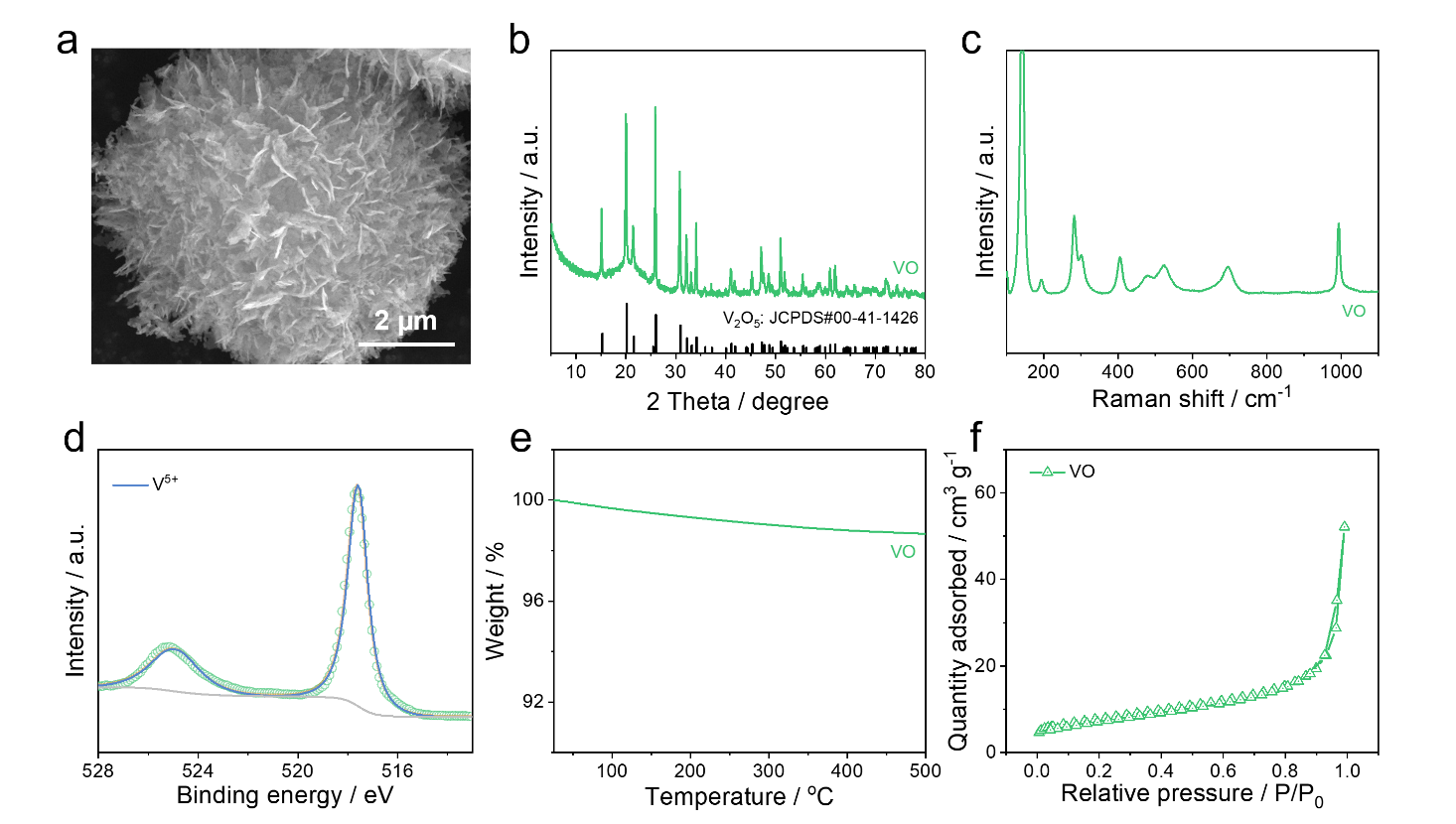


**Fig. S4** Characterization of VO: (**a**) SEM image. (**b**) XRD pattern. (**c**) Raman spectrum. (**d**) V 2p XPS spectra. (**e**) TGA curve. (**f**) N_2_ adsorption-desorption isotherm

To remove the interlayer crystalline water in HVO, it was annealed in air at 350 °C for 3 hours to obtain VO. The SEM image of VO is shown in Fig. S4a, where the flower-like morphology of the material is well preserved after going through the annealing process. All peaks in the XRD spectrum can be accurately matched to the standard data for orthogonal V_2_O_5_ (JCPDS#00-041-1426), and no additional diffraction peaks are detected (Fig. S4b). The Raman spectra can correspond to the characteristic modes of V_2_O_5_ (Fig. S4c). The XPS spectrum of V 2p is shown in Fig. S4d, vanadium in the VO is in the oxidation state of V^5+^. The TGA curve shows that as the temperature increases, VO undergoes only a slight weight loss, indicating that the interlayer crystalline water has been effectively removed (Fig. S4e). N_2_ adsorption-desorption isotherm of VO is very similar to that of HVO, both exhibiting a high specific surface area (Fig. S4f).


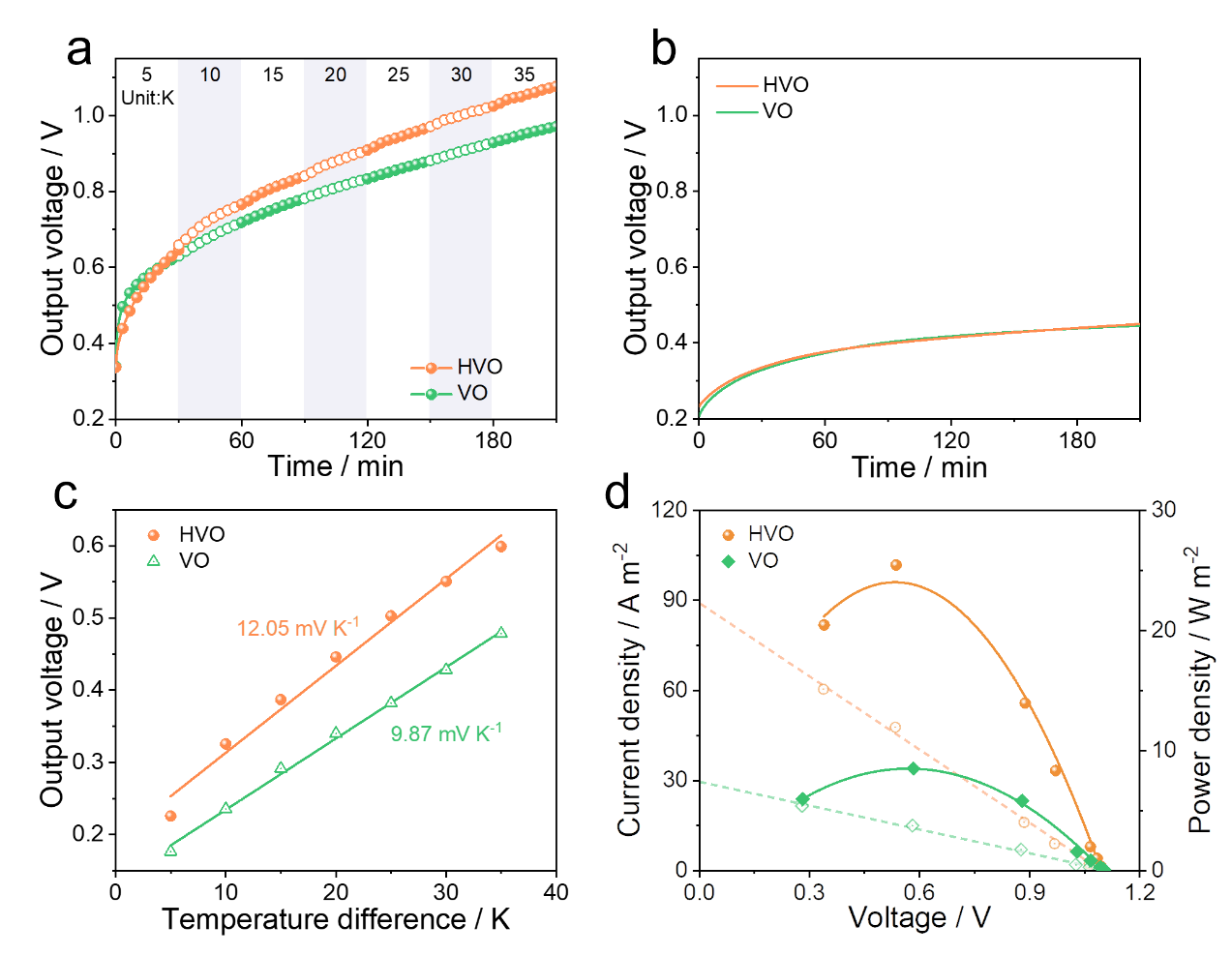


**Fig. S5** (**a**) Voltage variations at different temperature differences. (**b**) Self-charging curves. (**c**) Thermopower fitting plots. (**d**) Plots of power and current densities under various load resistances

To clarify whether the interlayer crystalline water of crystallization has an enhancement effect on the thermoelectric performance of V_2_O_5_, the low-grade heat conversion performance was tested in an H-type non-isothermal electrolytic cell utilizing the HVO and VO electrodes as cathode at the hot side, respectively, and matched with a Zn metal anode placed at the cold side, using a 0.5 M (NH_4_)_2_SO_4_ + 0.25 M ZnSO_4_ hybrid solution as the electrolyte.

The thermal charging curves of HVO∥Zn and VO∥Zn thermal charging cells are illustrated in Fig. S5a. The HVO∥Zn thermal charging cell shows a significantly faster voltage rise and a higher output voltage. When the temperature difference is 35 K, the maximum output voltages of HVO∥Zn and VO∥Zn thermal charging cells are 1.077 and 0.971 V, respectively, showing the great potential of the vanadium-based system for application. It should be noted that such a high output voltage is jointly caused by the thermal charging voltage and the electrochemical self-charging voltage. Fig. S5b shows the self-charging curve without an applied temperature difference. It can be observed that the voltage rises slowly, and after the same duration, the final self-charging voltage is approximately 0.45 V. The maximum pure thermal-induced voltage of the HVO∥Zn and VO∥Zn thermal charging cells was calculated to be 0.6 and 0.48 V. As shown in Fig. S5c, the thermopower of the HVO∥Zn and VO∥Zn thermal charging cells are 9.87 and 12.05 mV K⁻¹, respectively. Introducing interlayer crystalline water significantly enhances the low-grade heat conversion capability of V_2_O_5_.To investigate the differences in energy output capability of different systems, a series of resistors (100 kΩ-50 Ω) were chosen to perform discharge experiments on the thermal charged cells. As shown in Fig. S5d, the HVO∥Zn thermal charging cell exhibits excellent energy output performance, with a maximum power density of 24 W m^–2^ and a response current density of 89.8 A m^–2^, which is much higher than 8.5 W m^–2^ and 29.5 A m^–2^ of the VO∥Zn thermal charging cell.

This significant performance enhancement can be attributed to the unique structural characteristics of the HVO material. The presence of interlayer crystalline water provides a larger interlayer spacing, offering more space for ion migration and transport, thereby reducing the resistance to ion movement within the material. Additionally, the interlayer water can participate in the diffusion process of NH₄⁺, allowing for more effective ion migration and charge transfer during the thermoelectrochemical reaction, thus improving the system's thermoelectric conversion efficiency. In contrast, the smaller interlayer spacing of the VO material limits the speed of ion migration, resulting in inferior performance in energy conversion and thermoelectric response speed.


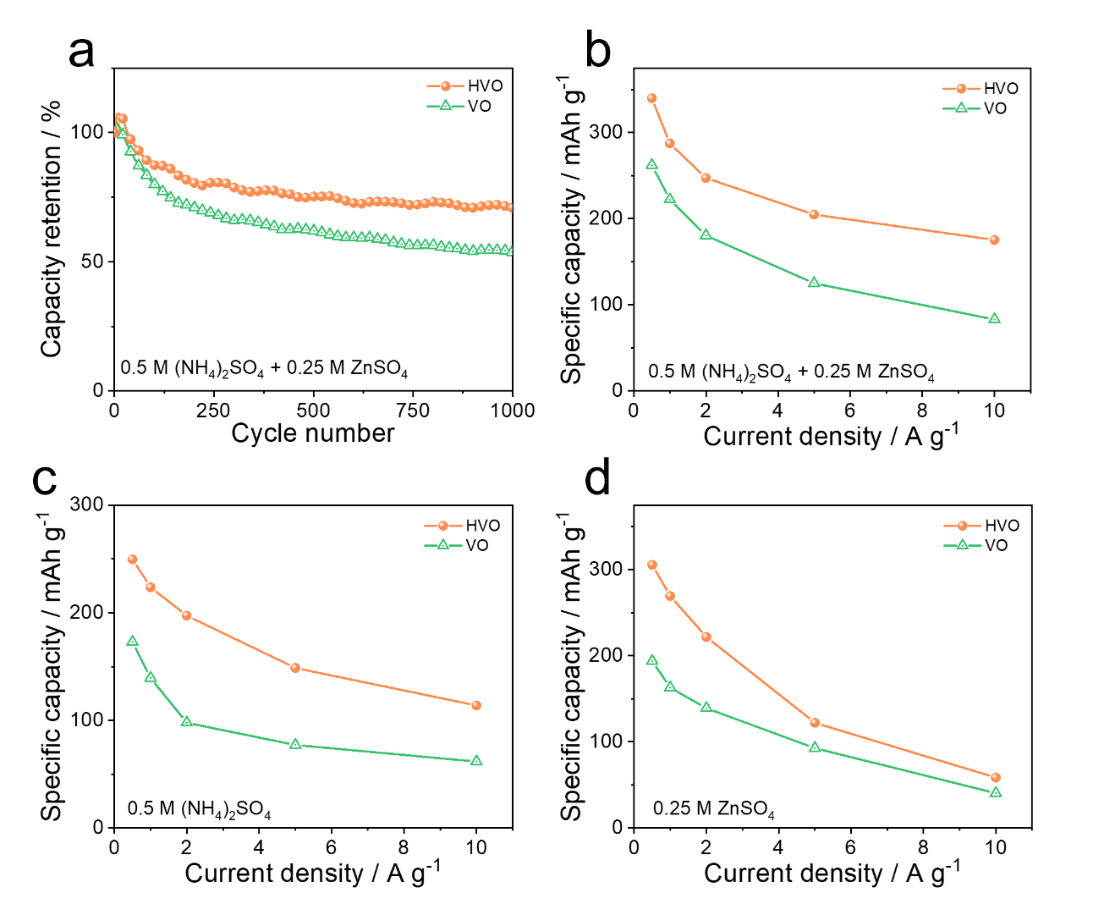


**Fig. S6** (**a**) Cycling stability comparison of HVO and VO electrodes in hybrid electrolyte. Rate capability comparison of HVO and VO electrodes in (**b**) hybrid electrolyte, (**c**) NH4⁺ electrolyte and (**d**) Zn²⁺ electrolyte

The interlayer crystal water in HVO plays multiple essential roles in enhancing the material's performance. First, the water molecules effectively expand the interlayer spacing in HVO, providing more spacious channels for ion transport. Second, these water molecules serve as structural pillars that stabilize the layered framework during repeated ion insertion/extraction cycles, as evidenced by the better cycling stability of HVO (Fig. S6a). When tested in the same hybrid ion electrolyte, VO exhibits substantially inferior capacity and rate performance compared to HVO, highlighting the essential function of crystalline water in facilitating ion transport (Fig. S6b).

Rate performance tests in both ammonium-ion and zinc-ion electrolytes consistently demonstrate HVO's superiority over VO. While the hydrogen-bond facilitation is particularly effective for NH₄⁺ due to its specific chemical nature, the expanded interlayer spacing also significantly benefits Zn²⁺ diffusion by reducing steric hindrance (Fig. S6c, d). The water molecules create a more favourable environment for ion transport regardless of the ion species.


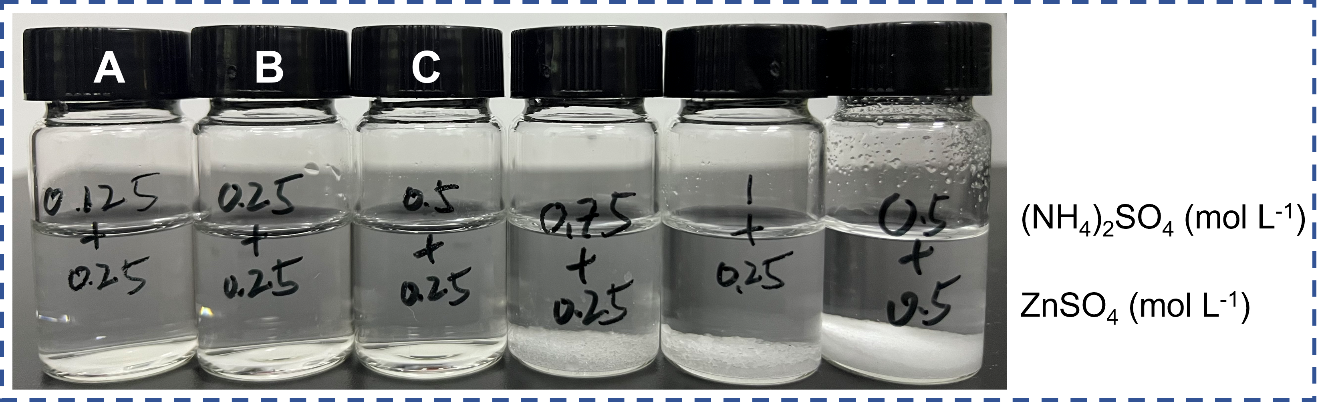


**Fig. S7** Comparison of solubility of hybrid-ion electrolytes with different ratios of Zn^2+^/NH_4_^+^

Several electrolytes with different ratios were designed in this work for preliminary screening. Their concentrations of ZnSO_4_ and (NH_4_)_2_SO_4_ were 0.25 M + 0.125 M, 0.25 M + 0.25 M, 0.25 M + 0.5 M, 0.25 M + 0.75 M, 0.25 M + 1 M, and 0.5 M + 0.5 M. As shown in Fig. S7 the latter three supersaturated solutions (0.25 M + 0.75 M, 0.25 M + 1 M, and 0.5 M + 0.5 M) were first ruled out as they showed significant precipitation and incomplete dissolution at room temperature (~25 °C).


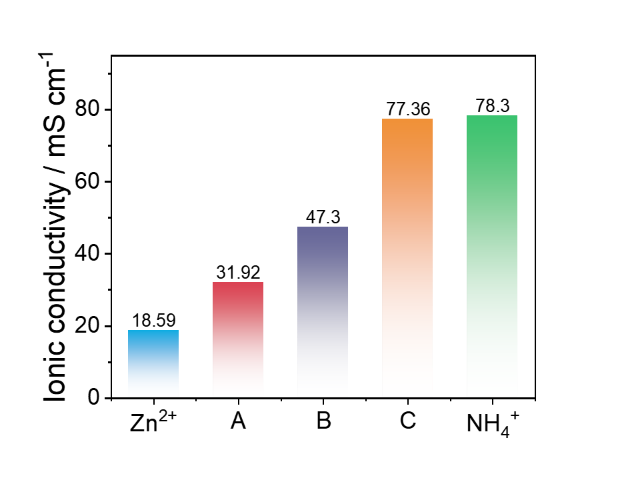


**Fig. S8** Ionic conductivities of different electrolytes, where A, B, and C represent 0.25 M ZnSO_4_ + 0.125, 0.25, and 0.5 M (NH_4_)_2_SO_4_ solutions, respectively; Zn^2+^ and NH_4_^+^ represent 0.25 M ZnSO_4_ solution and 0.5 M (NH_4_)_2_SO_4_ solution, respectively

The ionic conductivity of the electrolyte can reflect the diffusion rate of ions in different electrolytes to some extent. As shown in Fig. S8, the ionic conductivities of the mixed solutions of 0.25 M + 0.125 M, 0.25 M + 0.25 M, and 0.25 M + 0.5 M were 31.92, 47.3, and 77.36 mS cm^–1^, respectively, in addition to the ionic conductivities of the 0.25 M ZnSO_4_ and 0.5 M (NH_4_)_2_SO_4_ solutions of 18.59 and 78.3 mS cm^–1^. Since the hydrated ionic radius and ionic mass of NH_4_^+^ are smaller than that of Zn^2+^, it has a faster diffusion rate in aqueous solution, which makes the ionic conductivity of (NH_4_)_2_SO_4_ solution much larger than that of ZnSO_4_ solution. With the increase of the proportion of (NH_4_)_2_SO_4_ solution in the mixed electrolyte, the ionic conductivity of the mixed solution shows an obvious increasing trend, which indicates that the addition of NH_4_^+^ effectively improves the ionic conductivity of the hybrid-ion electrolyte and increases the total mobility of ions in the electrolyte, which is of great significance to improve the thermoelectric conversion efficiency and response speed of the system.


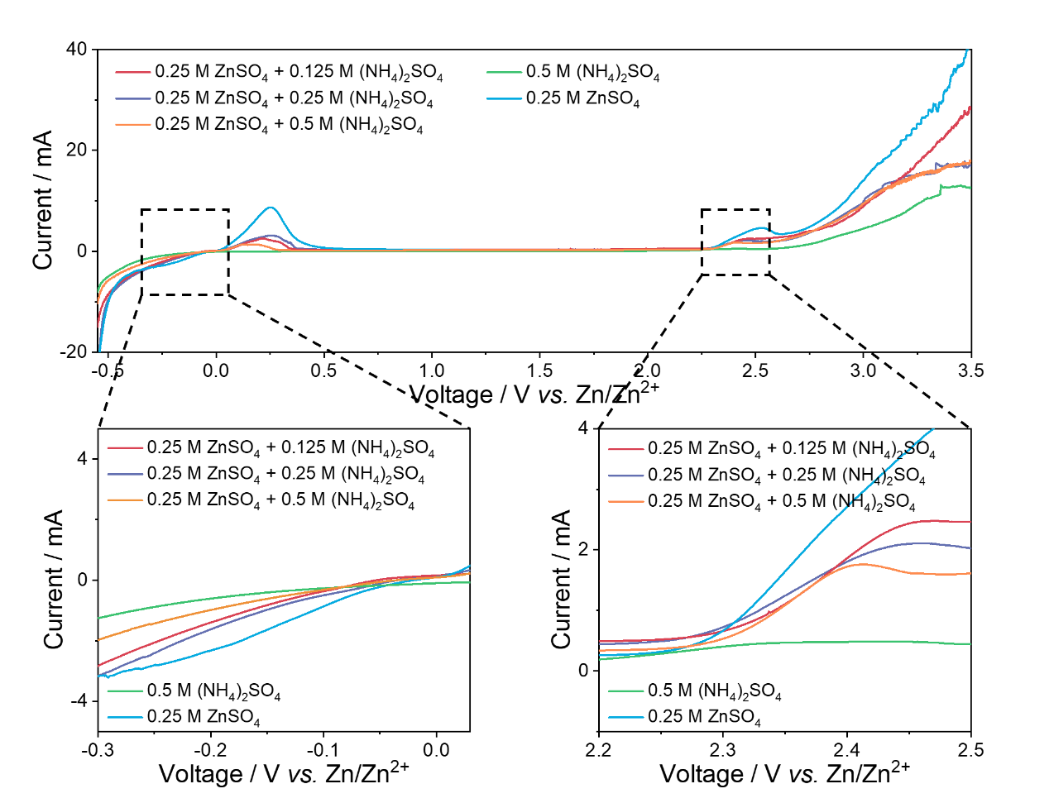


**Fig. S9** LSV profiles of different electrolytes in Zn∥SS cells with a scan rate of 5 mV s^−1^

Further, the electrochemical stability of different ratios of electrolytes was tested in Zn∥stainless steel (SS) asymmetric cells using linear scanning curves (LSV). As shown in Fig. S9, the electrochemical window of the 0.5 M (NH_4_)_2_SO_4_ solution is significantly wider than that of the more 0.25 M ZnSO_4_ solution, and the electrochemical window gradually widens as the ratio of the (NH_4_)_2_SO_4_ solution increases. Among them, the 0.25 M ZnSO_4_ + 0.5 M (NH_4_)_2_SO_4_ electrolyte exhibited the widest electrochemical window (>2.2 V) among the three mixed solutions, showing excellent electrochemical stability.


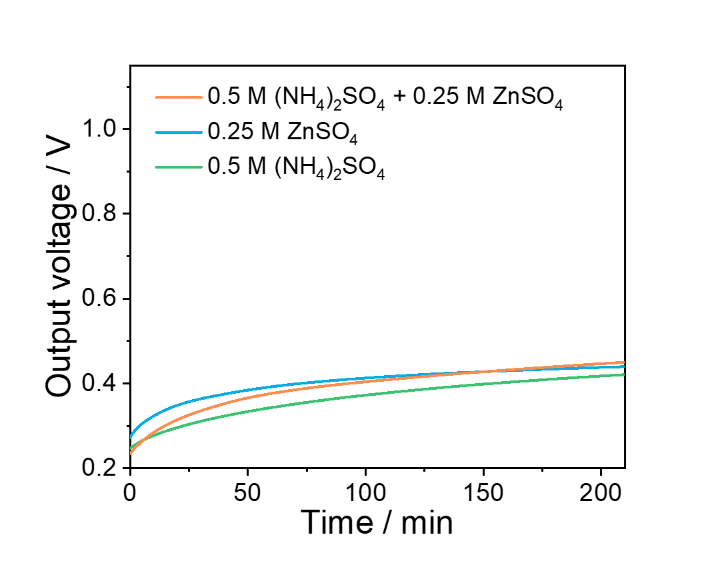


**Fig. S10** Self-charging curves of three thermal charging cells


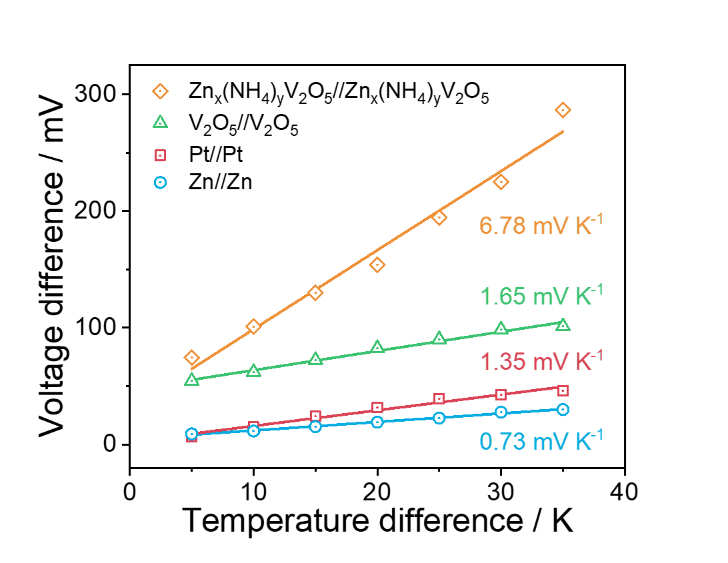


**Fig. S11** Temperature coefficients of various systems

To deeply understand the contribution of thermal diffusion and thermoextraction processes to the thermopower of HTCC, as shown in Fig. S11, we assembled four symmetric non-isothermal H-type electrolytic cells. The total thermopower value can be divided into four components as shown in Eq. S1:

$S_{i}=\alpha_{{{Zn}_{x}({NH}_{4})}_{y}V_{2}O_{5}/V_{2}O_{5}}+\alpha_{Zn/{Zn}^{2+}}+S_{td(electrolyte)}+S_{td(electrode)}$ (S1)

The thermal charging response of Pt∥Pt (1.35 mV K^−1^) represents the thermodiffusion of ions in the electrolyte. Notably, when using Zn as electrodes, the value is 0.73 mV K^−1^, which indicates the deposition/stripping process of Zn^2+^ on the Zn foil anode. Meanwhile, the thermopower from the reaction between Zn_x_(NH_4_)_y_V_2_O_5_ and V_2_O_5_ is about 5.13 mV K^−1^, distinguished by the values obtained by Zn_x_(NH_4_)_y_V_2_O_5_∥Zn_x_(NH_4_)_y_V_2_O_5_ and V_2_O_5_∥V_2_O_5_.


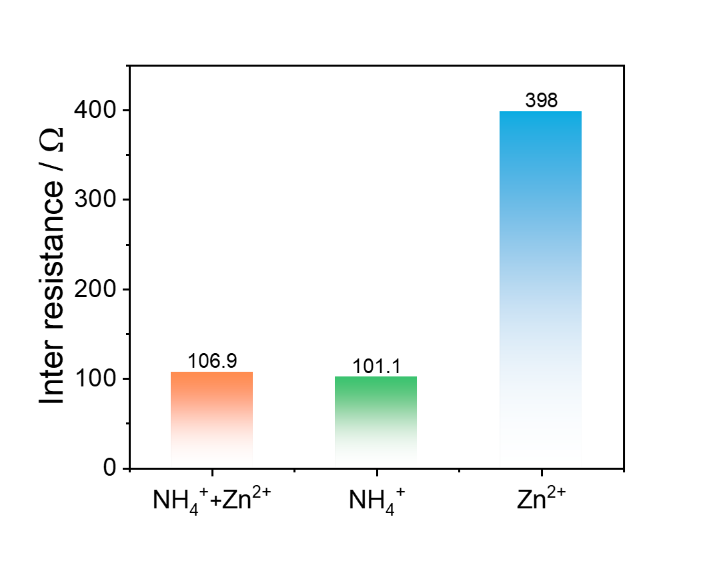


**Fig. S12** Inter resistance of three thermal charging cells


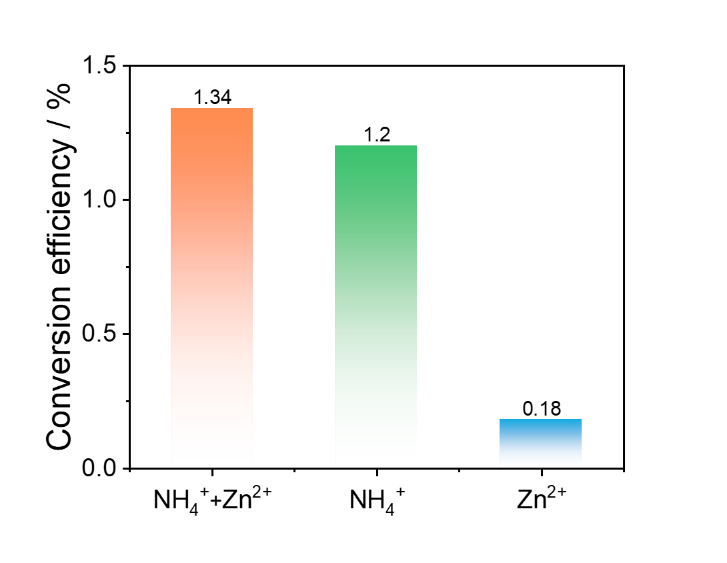


**Fig. S13** Energy conversion efficiency of three thermal charging cells


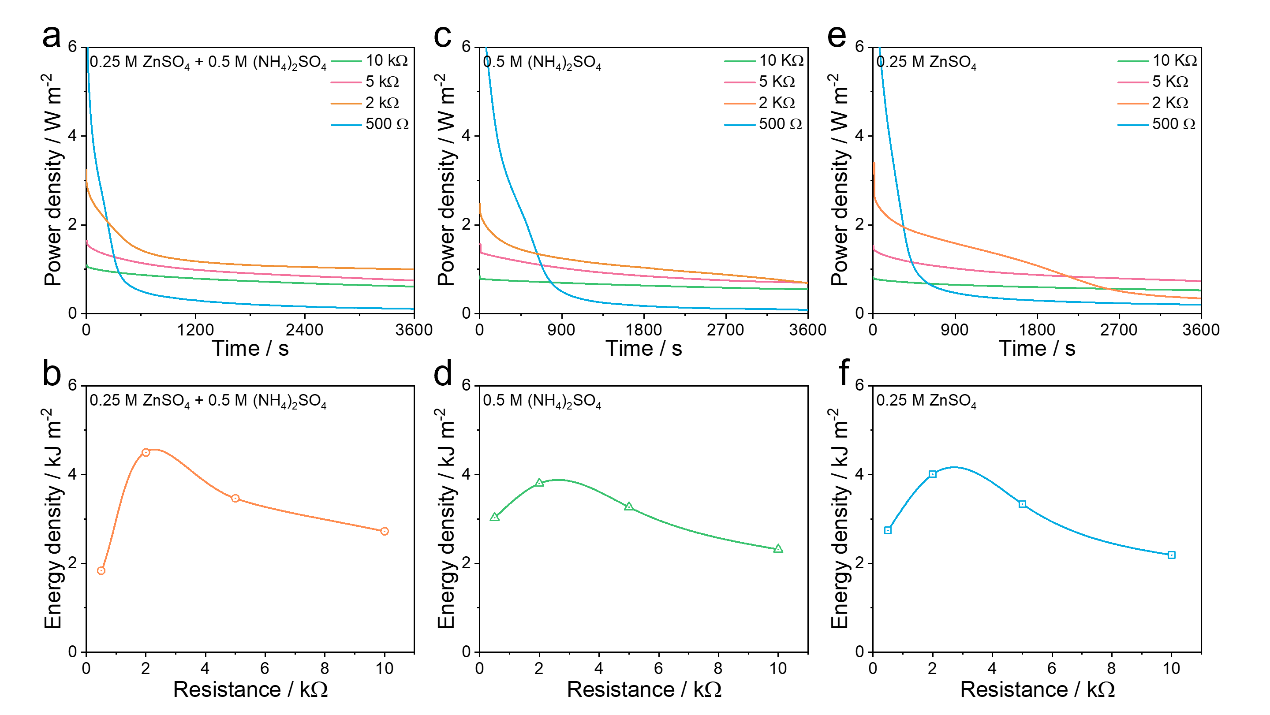


**Fig. S14** Power change under continuous discharge operation with different external resistors at 35 K temperature difference and the corresponding energy densities: (**a, b**) HTCC, (**c, d**) ATCC, (**e, f**) ZTCC

**
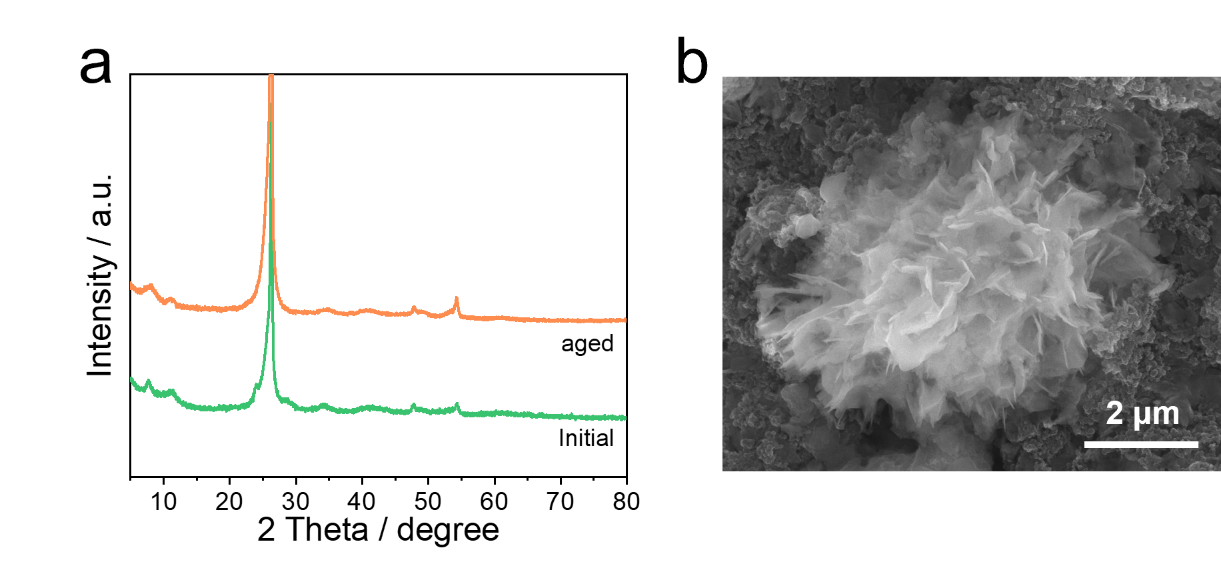
**

**Fig. S15** Structural characterization of the HVO cathode after the 72-hour test: (**a**) XRD patterns and (**b**) SEM image


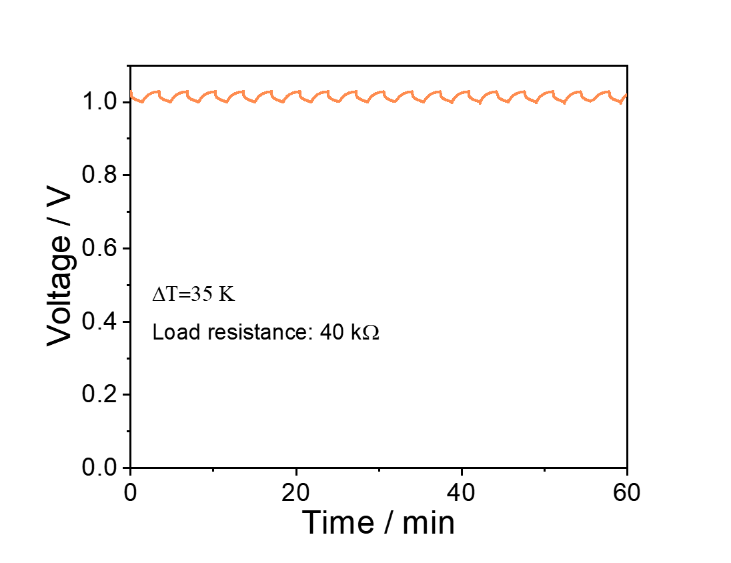


**Fig. S16** Capacity test after prolonged operation

Regarding the cathode structural evolution, XRD characterization of the HVO cathode tested for 72 hours confirms the well-maintained layered crystal structure without detectable phase transformation (Fig. S15a). SEM examination further reveals complete preservation of the original nanoflower morphology (Fig. S15b). These results collectively demonstrate the remarkable structural stability and outstanding reversibility of the Zn²⁺/NH₄⁺ co-insertion/extraction process.

As shown in the Fig. S16, we conducted systematic charge-discharge tests on the device after 72 hours of continuous operation. The results demonstrate that the device maintains its capacity without significant decay after prolonged operation, showing excellent capacity retention. This finding, consistent with the well-preserved cathode structure, collectively confirms the outstanding long-term operational stability of our thermal charging cell.


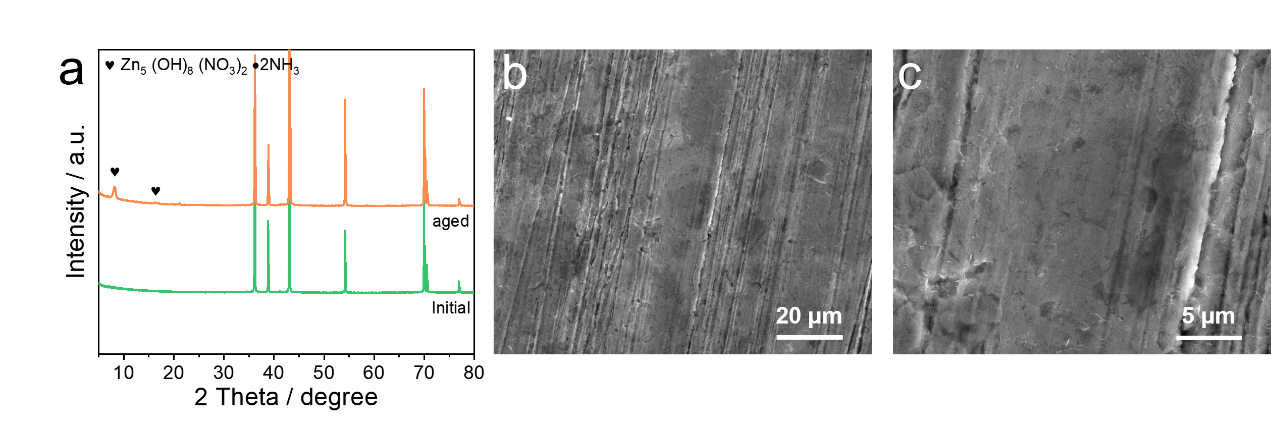


**Fig. S17** Characterization of the Zn anode after prolonged operation: (**a**) XRD patterns and (**b**) SEM images

We performed post-cycling characterization of the Zn anode after prolonged operation in the hybrid electrolyte. XRD analysis reveals the presence of minor Zn_5_(OH)_8_(NO_3_)_2_·2NH_3_ byproducts, though their intensity remains relatively weak, indicating limited formation (Fig. S17a). Corresponding SEM observations revealed slight zinc dendrite formation on the Zn anode surface, but the overall morphology of the electrode remained largely intact, with no severe corrosion observed (Fig. S17b-c). These results suggest that while some side reactions occur at the Zn electrode, their extent is not sufficient to cause significant performance degradation.


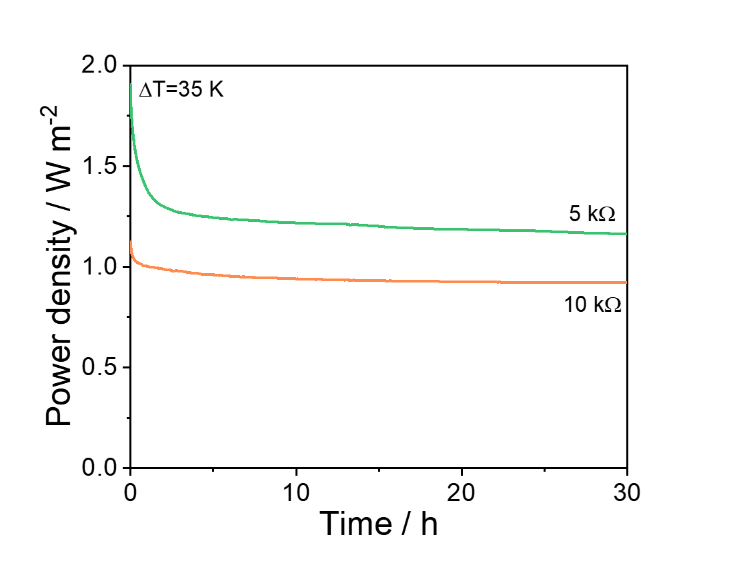


**Fig. S18** Long-term discharge of HTCC at 35 K temperature difference

Figure S18 demonstrates the device's operational stability under high-power matched loads. When tested with load resistances of 10 kΩ and 5 kΩ, the HTCC maintains stable operation for over 30 hours, with power levels sustained at approximately 0.92 W m⁻² and 1.2 W m⁻², respectively. These results confirm the device's capability for prolonged operation at practically relevant power outputs.


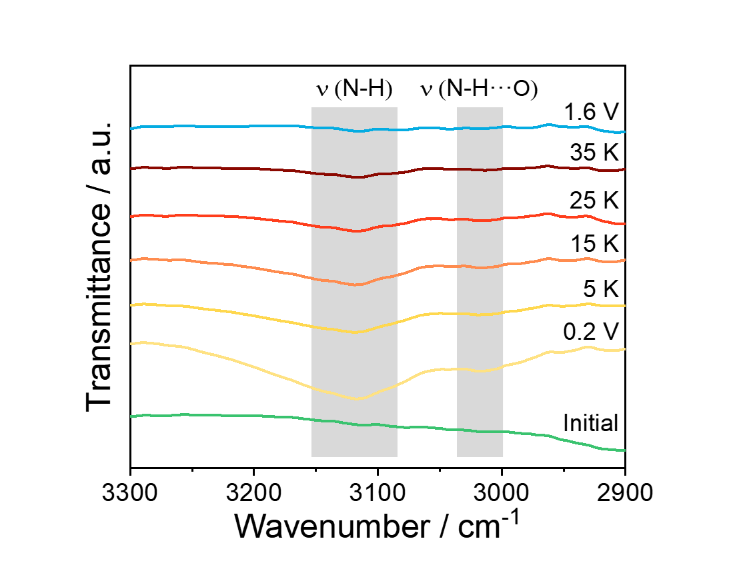


**Fig. S19** The ex situ FT-IR patterns of HVO in HTCC


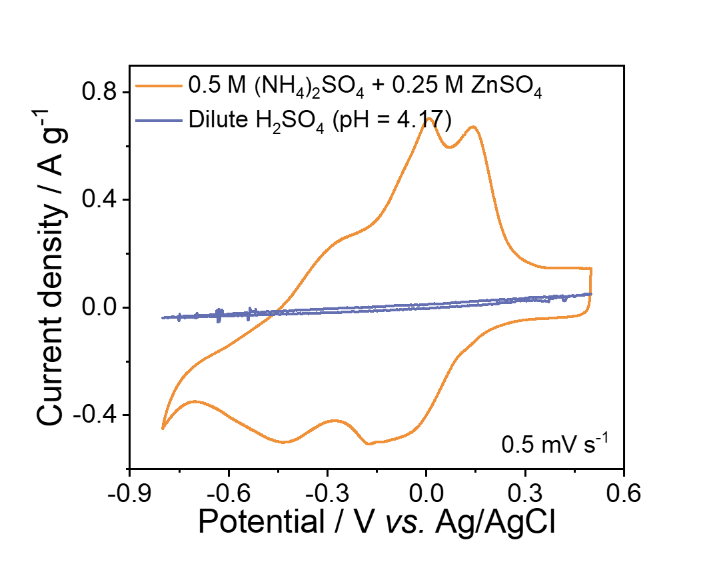


**Fig. S20** CV curves of HVO in dilute H_2_SO_4_ (pH = 4.17)
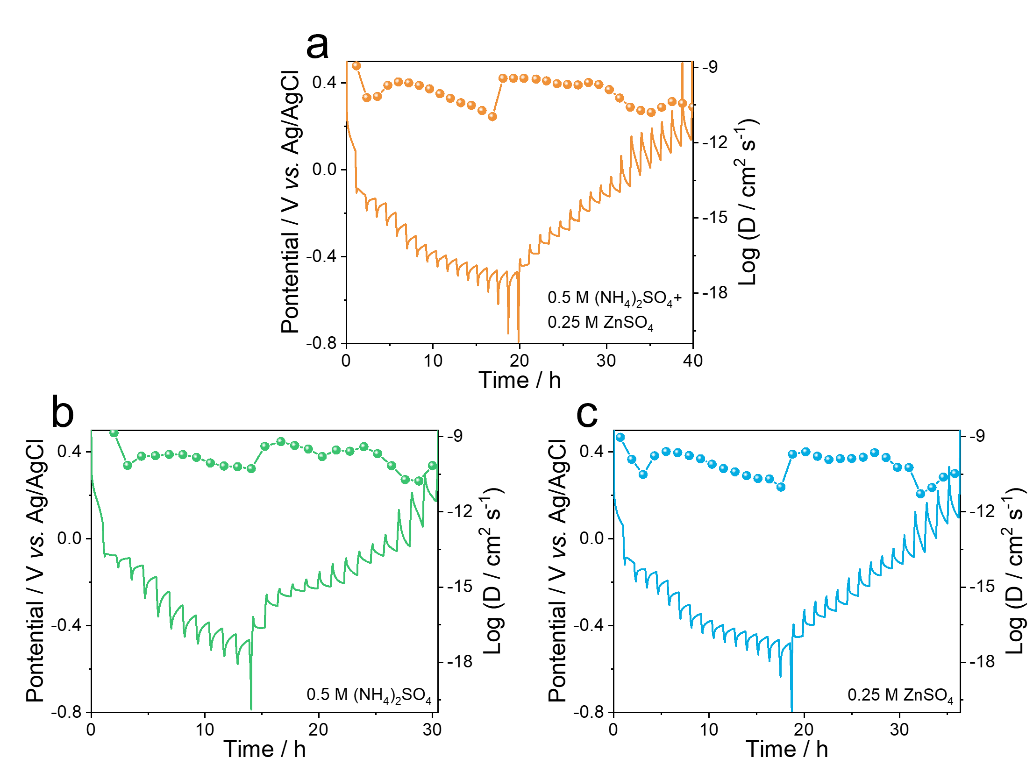


**Fig. S21** GITT curves and ion diffusion coefficient of HVO in different electrolytes

The value of ionic diffusion coefficients can be calculated by simplified Fick’s second law:

$D=\frac{4}{\pi\tau}\left( \frac{m_{b}V_{m}}{M_{b}S} \right)^{2}\left( \frac{{\Delta E}_{s}}{\tau\left( {dE_{\tau}}/{d\sqrt{\tau}} \right)} \right)^{2}\left( \tau\ll{L^{2}}/D \right)$ (S2)

where $m_{b}$ represents the active substance loading, $M_{b}$ represents the molar mass of the sample, $V_{m}$ represents the molar volume of the sample, $S$ is the geometric area of the electrode, and $S$ is the electrode thickness. In general, the potential is linearly related to $\tau^{1/2}$, then Eq. S2 can be simplified to the Eq. S3:

$D=\frac{4}{\pi\tau}\left( \frac{n_{m}V_{m}}{S} \right)^{2}\left( \frac{{\Delta E}_{s}}{{\Delta E}_{\tau}} \right)^{2}$ (S3)


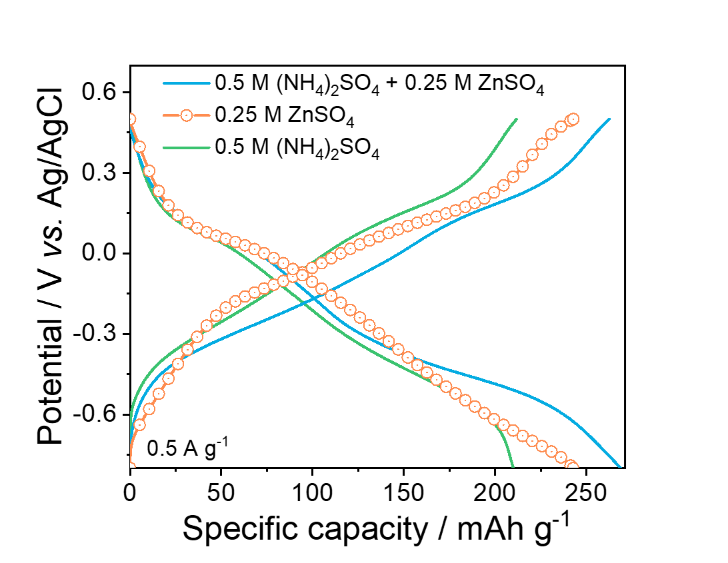


**Fig. S22** GCD curves of HVO in different electrolytes at 0.5 A g^–1^


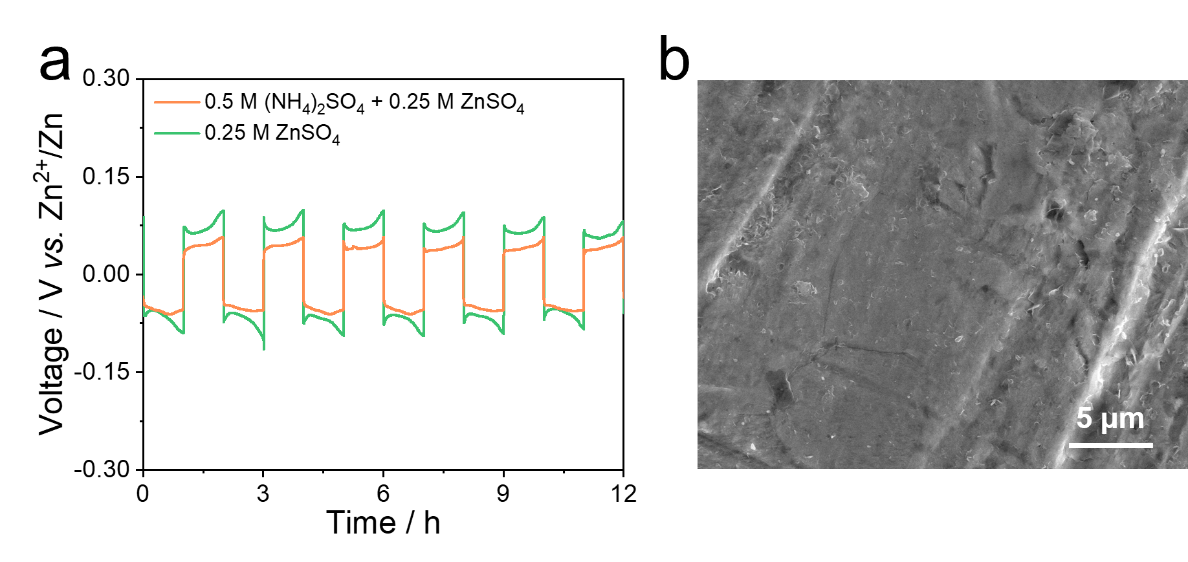


**Fig. S23** (**a**) Voltage profiles of Zn||Zn symmetric cells cycled at 0.5 mA cm⁻² in hybrid electrolyte and Zn²⁺ electrolyte. (**b**) SEM image of the Zn electrode after cycling in hybrid electrolyte

Test results from Zn||Zn symmetric electrodes indicate that the polarization voltage of the mixed electrolyte is lower than that of the electrolyte containing only Zn²⁺ (Fig. S23a). This observation suggests that NH₄⁺ ions promote more uniform zinc deposition through electrostatic interactions. SEM characterization of the Zn electrode after 24 hours of cycling shows well-maintained morphology without noticeable zinc dendrite formation, confirming the electrode's structural integrity under prolonged operation (Fig. S23b).


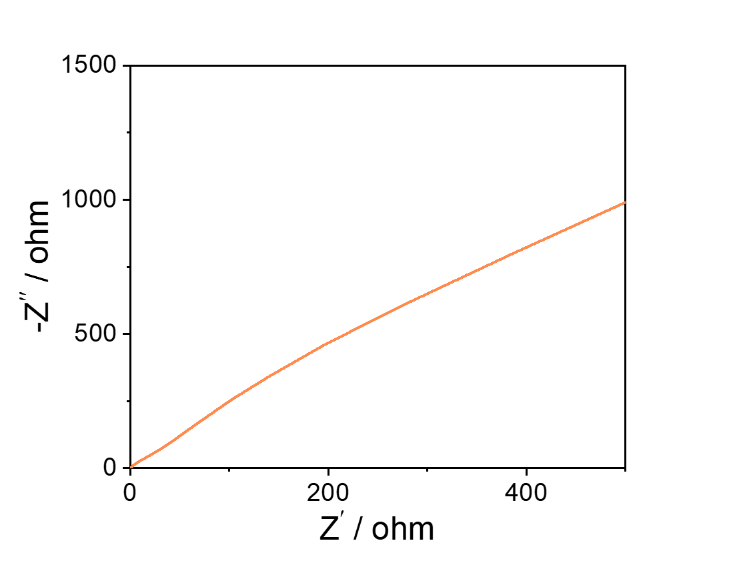


**Fig. S24** EIS spectra of PAM hydrogel electrolyte


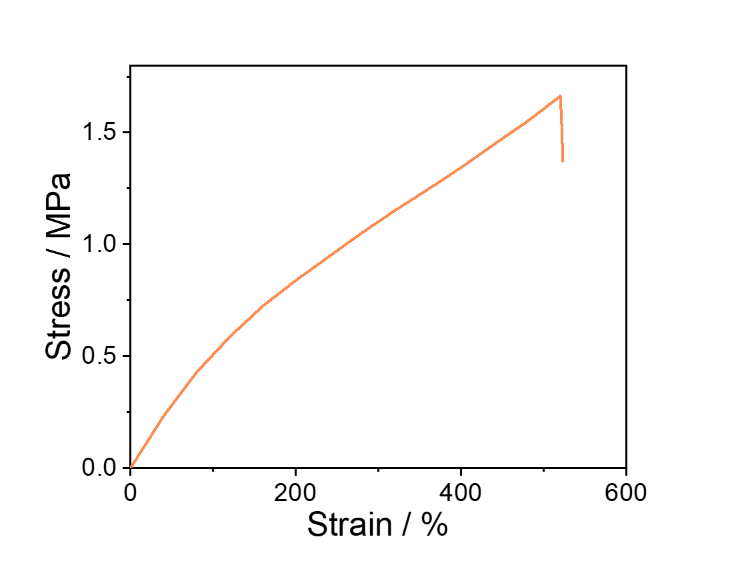


**Fig. S25** Tensile stress-strain curve of PAM hydrogel electrolyte

The thermal conductivity of the hydrogel electrolyte was measured based on the steady-state method (heat conservation law). The hydrogel was heated at a specific temperature for 1 hour, and the thermal distribution within the hydrogel was determined using a Fourier-transform infrared thermal imager (FLIR T630sc). The thermal conductivity (κ) was calculated using the Eq. S4:

$\kappa=\frac{Q\times L}{A\times\Delta T}$ (S4)

where Q is the applied heat flux, L is the thickness of the hydrogel sample, A is the cross-sectional area, and ΔT is the temperature difference across the sample. Calculations reveal that the PAM hydrogel exhibits a thermal conductivity of 0.52 W m⁻¹ K⁻¹, which is favourable for maintaining the necessary temperature gradient across the device.

The ionic conductivity (σ) is tested by sandwiching the hydrogel electrolytes between two stainless teel plates to form a coin-type cell on the Biologic VMP-300 workstation. After obtaining the corresponding impedance value, it is further calculated by using Eq. S5.

$\sigma=\frac{d}{R\times S}$ (S5)

The d and S represent the thickness and area of hydrogel electrolyte, and R refers to the ionic impedance (Fig. S24). Calculations reveal that the ionic conductivity of PAM hydrogel is 22.3 mS cm^–1^ at room temperature, ensuring efficient ion transport.

Furthermore, Fig. S25 demonstrates that the hydrogel possesses good elasticity and can withstand repeated deformation, which is crucial for the durability of flexible devices.


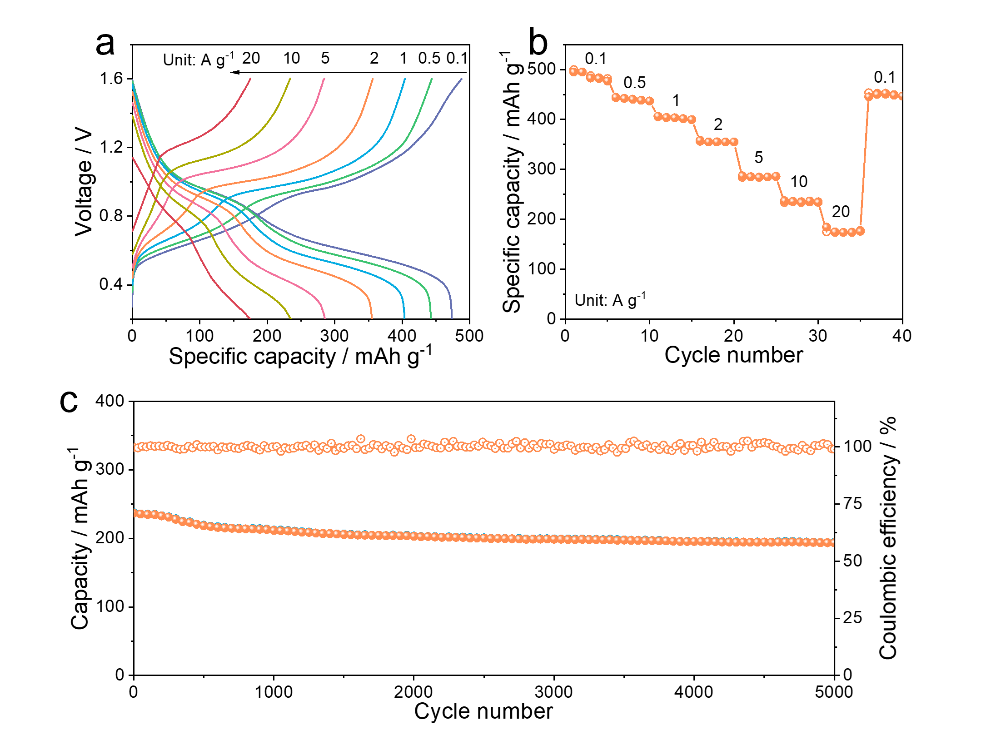


**Fig. S26** (**a**) GCD curves of HIB at different current densities. (**b**) Rate performance of HIB. (**c**) Cycling stability performance of HIB at 10 A g^−1^

To assess the energy storage capacity of the constructed hybrid ion system, coin-type Zn^2+^/NH_4_^+^ hybrid-ion batteries (HIBs) were reasonably constructed. As shown in Fig. S26a and b, the HIB exhibits excellent rate capability, in which 495.3, 442.2, 404.1, 353.4, 355.6, 284.6, 235.8, and 174.2 mAh g^−1^ can be recorded at 0.1, 0.2, 0.5, 1, 2, 5, 10, and 20 A g^−1^, respectively. Especially under the high current density of 20 A g^−1^, the HIB can maintain GCD curve shapes similar to those at low current densities, which further demonstrates excellent electrochemical reversibility. Notably, when the current density returned to the initial 0.1 A g^−1^, the specific capacity recovered to 452.5 mAh g^−1^, achieving a retention rate of 91.2%. As depicted in Fig. S26c, even at a high current density of 10 A g^−1^, the HIB demonstrates remarkable stability. It retains a specific capacity of 192 mA h g^−1^ over 5000 cycles, with an 80% capacity retention rate, and maintains a Coulombic efficiency of 100% throughout.

**Table S1** Parameters for energy conversion efficiency calculation of three thermal charging cells

|  | NH_4_^+^+Zn^2+^ | NH_4_^+^ | Zn^2+^ |
| --- | --- | --- | --- |
| Thermopower / mV K^−1^ | 12.05 | 11.1 | 8.5 |
| Cold side temperature / K | 298.15 | 298.15 | 298.15 |
| Hot side temperature / K | 333.15 | 333.15 | 333.15 |
| Inter-electrode spacing / m | 0.07 | 0.07 | 0.07 |
| Thermal conductivity / W m^−1^ K^−1^ | 0.55 | 0.55 | 0.55 |
| Cross sectional area of the cell / m^2^ | 0.000113 | 0.000113 | 0.000113 |
| Inter resistance obtained by voltage-current plot / Ω | 106.903 | 101.137 | 398.033 |
